# Clinical Remission of Sight-Threatening Non-Infectious Uveitis Is Characterized by an Upregulation of Peripheral T-Regulatory Cell Polarized Towards T-bet and TIGIT

**DOI:** 10.3389/fimmu.2018.00907

**Published:** 2018-05-03

**Authors:** Rose M. Gilbert, Xiaozhe Zhang, Robert D. Sampson, Michael R. Ehrenstein, Dao X. Nguyen, Mahid Chaudhry, Charles Mein, Nadiya Mahmud, Grazyna Galatowicz, Oren Tomkins-Netzer, Virginia L. Calder, Sue Lightman

**Affiliations:** ^1^Ocular Immunology, Institute of Ophthalmology, University College London (UCL), London, United Kingdom; ^2^Moorfields Eye Hospital NHS Foundation Trust, London, United Kingdom; ^3^Flow Cytometry Core Facility, Institute of Ophthalmology, University College London (UCL), London, United Kingdom; ^4^Division of Medicine, Centre for Rheumatology, University College London (UCL), London, United Kingdom; ^5^Genome Centre, Barts and the London School of Medicine and Dentistry, Queen Mary University of London, London, United Kingdom

**Keywords:** uveitis, T-regulatory cells, TIGIT, T-bet, ocular inflammation, remission, Th1, Th17

## Abstract

**Background:**

Non-infectious uveitis can cause chronic relapsing and remitting ocular inflammation, which may require high dose systemic immunosuppression to prevent severe sight loss. It has been classically described as an autoimmune disease, mediated by pro-inflammatory Th1 and Th17 T-cell subsets. Studies suggest that natural immunosuppressive CD4^+^CD25^+^FoxP3^+^ T-regulatory cells (Tregs) are involved in resolution of inflammation and may be involved in the maintenance of clinical remission.

**Objective:**

To investigate whether there is a peripheral blood immunoregulatory phenotype associated with clinical remission of sight-threatening non-infectious uveitis by comparing peripheral blood levels of Treg, Th1, and Th17, and associated DNA methylation and cytokine levels in patients with active uveitic disease, control subjects and patients (with previously active disease) in clinical remission induced by immunosuppressive drugs.

**Methods:**

Isolated peripheral blood mononuclear cells (PBMC) from peripheral blood samples from prospectively recruited subjects were analyzed by flow cytometry for CD3, CD4, FoxP3, TIGIT, T-bet, and related orphan receptor γt. Epigenetic DNA methylation levels of FOXP3 Treg-specific demethylated region (TSDR), FOXP3 promoter, TBX21, RORC2, and TIGIT loci were determined in cryopreserved PBMC using a next-generation sequencing approach. Related cytokines were measured in blood sera. Functional suppressive capacity of Treg was assessed using T-cell proliferation assays.

**Results:**

Fifty patients with uveitis (intermediate, posterior, and panuveitis) and 10 control subjects were recruited. The frequency of CD4^+^CD25^+^FoxP3^+^ Treg, TIGIT^+^ Treg, and T-bet^+^ Treg and the ratio of Treg to Th1 were significantly higher in remission patients compared with patients with active uveitic disease; and TIGIT^+^ Tregs were a significant predictor of clinical remission. Treg from patients in clinical remission demonstrated a high level of *in vitro* suppressive function compared with Treg from control subjects and from patients with untreated active disease. PBMC from patients in clinical remission had significantly lower methylation levels at the FOXP3 TSDR, FOXP3 promoter, and TIGIT loci and higher levels at RORC loci than those with active disease. Clinical remission was also associated with significantly higher serum levels of transforming growth factor β and IL-10, which positively correlated with Treg levels, and lower serum levels of IFNγ, IL-17A, and IL-22 compared with patients with active disease.

**Conclusion:**

Clinical remission of sight-threatening non-infectious uveitis has an immunoregulatory phenotype characterized by upregulation of peripheral Treg, polarized toward T-bet and TIGIT. These findings may assist with individualized therapy of uveitis, by informing whether drug therapy has induced phenotypically stable Treg associated with long-term clinical remission.

## Introduction

Uveitis, defined as inflammation of the uveal inner layer of the eye, is the fifth commonest cause of visual loss in the developed world, accounting for about 10–15% of the cases of total blindness (World Health Authority definition) and up to 20% of legal blindness (1). Incidences vary between 38 and 200 per 100,000 (2). Uveitis can affect people of all ages but occurs most frequently in the working age population (20–50 years), with 35% affected remaining visually disabled (3). In the majority of cases, the inflammation in uveitis is non-infectious, of idiopathic and presumed autoimmune etiology. This type of disease may follow a relapsing, remitting course, which is clinically challenging to manage. Severe cases may require treatment with high dose oral corticosteroid drugs to minimize irreversible damage to the retina and visually important structures, which result in sight loss. Uveitis is a clinically heterogenous disease: at least 150 disorders are known to be associated with intraocular inflammation, although these disorders are individually rare. The Standardization of Uveitis Nomenclature (SUN) working group, therefore, developed clinical criteria to classify uveitis based on the anatomical localization of the inflammation (4). These criteria are useful but they do not define specific disease entities, phases of disease or include immunological biomarkers.

Our understanding of the immune mechanisms involved in non-infectious uveitis has derived from animal studies of experimental autoimmune uveitis (EAU) (5). Inflammation in EAU is mediated by Th1 and Th17 subsets of self-reactive CD4 T-lymphocytes. The Th1 and Th17 lineages are characterized by expression of T-bet (*Tbx21*) and retinoic-acid related orphan receptor (ROR) γ-t (*Rorc*) transcription factors and secretion of the “signature” pro-inflammatory cytokines IFNγ and IL-17, respectively. Phenotypically categorized CD4^+^CD25^+^FoxP3^+^ T-regulatory cells (Tregs) exist as a naturally occurring mechanism to suppress these autoreactive T-cells through production of anti-inflammatory cytokines such as IL-10 and transforming growth factor β (TGF-β) (6), in addition to many other mechanisms. Studies in rat models of EAU have shown that during resolution of the first acute attack of uveitis, the number of ocular Treg is increased (7). In those rats that went on to develop recurrent EAU, the suppressor function of Treg was found to be weaker (7). In murine models of EAU, a significantly increased frequency and immunoregulatory action of CD4^+^CD25^+^ Treg cells has been associated with the regression of EAU, suggesting that CD4^+^CD25^+^ Tregs are induced during EAU and may be involved in disease resolution (8). Further evidence in the mouse model demonstrates that retina-specific functionally suppressive FoxP3^+^ Tregs accumulate in inflamed eyes and persist for several months after disease remission (9). Depletion of Treg at the peak of uveitis delayed resolution and, following resolution where mice displayed a low grade chronic inflammation, Treg depletion precipitated disease relapse (9).

Many human studies have examined the role of Treg in systemic autoimmune diseases, where defects in Treg numbers and/or suppressive function have been found, in addition to imbalances between pro-inflammatory T-effectors and immunosuppressive Treg (10, 11). Patients with active uveitis have decreased peripheral blood levels of Tregs compared with healthy control subjects, with levels being significantly upregulated during disease remission (12, 13). Low or absent levels of FoxP3 mRNA are found in Tregs from patients with severe, recalcitrant uveitis (13). In rheumatoid arthritis (RA), Tregs recover their suppressive function after immunomodulating treatment with anti-tumor necrosis factor (TNF) α (11) monoclonal antibody (Ab) therapy. More specifically, the anti-TNFα agent, adalimumab, has been shown to restore immune tolerance in RA through expansion of functional Foxp3^+^ Treg cells, equipped to suppress Th17 effector cells (11, 14, 15). In clinical trials of systemically administered immunomodulating therapies in uveitis, induction of disease remission is associated with systemic upregulation of Tregs and modulation of cytokine production (16, 17). However, pharmacological effects on cytokines have also been demonstrated without a significant increase in the proportion of systemic FoxP3^+^ Treg levels (18). Taken as a whole, these studies suggest that the mechanism of disease remission in uveitis may involve upregulation of systemic Treg and/or restoration of their suppressive function, the effects of which may be mediated through cytokines. Furthermore, “functional” Treg seem to be important for preventing disease relapse and maintaining long-term clinical remission.

A lack of consensus regarding immunophenotypic markers for functionally suppressive Treg has meant that their status in human autoimmune disease has not been fully understood. Inflammatory environments induce T-cell plasticity (19) in which peripherally induced CD4^+^CD25^+^FoxP3^+^ Treg (pTregs) subsets may co-exist with more phenotypically stable thymically derived CD4^+^CD25^+^FoxP3^+^ Tregs (nTregs). Foxp3 is considered the Treg “master transcription factor” because it is critically required for Treg-cell development and function and for suppressing autoimmunity (20–23). However, it is also known that activated human T-cells may transiently upregulate FOXP3 and that these FoxP3^+^ T-cells are not effective at suppressing inflammation (24–27). Studies have revealed that a specific DNA hypomethylation pattern, the Treg-specific demethylated region (TSDR), a hypomethylated region within the FOXP3 gene, is associated with enhancement and stabilization of Foxp3 expression in Treg (28–31). In combination with CD25 and FoxP3 expression, the TSDR has allowed identification of Treg with a stable phenotype (32). The addition of a methyl group (–CH3) to DNA is a common epigenetic mechanism that cells use to switch genes “off.” Extensive methylation of cytosine in DNA is known to correlate with reduced gene transcription. DNA methylation is generally thought to play an essential role in T-cell function and failure to maintain DNA methylation patterns in mature T-cells has been implicated in the development of autoimmune disease (33).

A recently discovered co-inhibitory molecule, T cell immunoreceptor with Ig and ITIM domains (TIGIT), is expressed by Treg (34) and suppresses a range of immune cells. Studies show that TIGIT ligation directly inhibits T cell proliferation and cytokine production in CD4^+^ T cells (35). Furthermore, increased expression of TIGIT, which delineates Treg from activated effector T cells, has been associated with hypomethylation and FOXP3 binding at the TIGIT locus (36). Treg cells expressing TIGIT were found to selectively inhibit Th1 and Th17, but not Th2 responses (37). Since it is known that the Th1 and Th17 subsets are pivotal to the pathogenesis of autoimmune disease, TIGIT may be a biomarker of stable, functionally suppressive Treg in these diseases.

Treatment paradigms of systemic autoimmune diseases, such as RA and inflammatory bowel disease, have now evolved beyond partial symptom control (38). The goal of inducing and maintaining sustained biological remission, to improve long-term disease outcomes, has been described as a “treat-to-target” strategy (38). A working definition of sustained “deep remission,” which includes long-term biological remission and symptom control with defined patient outcomes, including no disease progression, has been proposed for Crohn’s disease (38, 39). “Clinical remission” in uveitis, however, has not been defined or investigated as an immunologically distinct phase of disease. Understanding the immunological mechanisms underlying disease remission, in addition to the focus on inhibiting inflammation, could have significant implications for clinical phenotyping of uveitis and determining the best therapeutic approach to induce remission for each patient.

In this study, we investigate the hypothesis that there is a peripheral blood immunoregulatory T-cell phenotype associated with sustained clinical remission, using a prospective study design with recruitment of 50 non-infectious uveitis patients (without identifiable systemic disease) and 10 control subjects. Clinical remission is defined as a 6-month period of remission without treatment, in patients with previously active relapsing and remitting disease, which clinically responded to systemic immunosuppressive therapy. We evaluate the frequency of CD4^+^CD25^+^FoxP3^+^ Treg and, additionally, the frequency of TIGIT^+^ Treg, which we propose to be a biomarker of functionally suppressive Treg. Co-expression of FoxP3, ROR γ-t, and T-bet, the ratio of Treg to putative T-effectors, and serum levels of cytokines associated with these T-cell subsets are also analyzed. Furthermore, we evaluate whether there is an epigenetic immune methylation pattern associated with clinical remission in uveitis, using a targeted next-generation sequencing (NGS) bisulfate sequencing approach toward T-cell genes of interest.

## Materials and Methods

### Patient Recruitment and Specimen Collection

A total of 50 patients with a diagnosis of idiopathic non-infectious uveitis were consecutively recruited to the study from Moorfields Eye Hospital, London, UK between November 2014 and December 2016. Inclusion criteria for all patients were as follows: age 18–59 years; a current or previous diagnosis of non-infectious intermediate, posterior or panuveitis as per SUN (4); and the absence of known associated infectious disease or systemic inflammatory disease. Eligibility for recruitment was further determined by uveitic disease activity. To meet the clinical criteria for disease “in clinical remission,” patients were required to have no signs of disease activity at the time of recruitment; have a previous diagnosis of active non-infectious chronic relapsing and remitting uveitis which was treated with oral corticosteroids ± second-line corticosteroid-sparing treatments before the period of clinical remission; and have discontinued all immunosuppressive treatment and remained “quiescent” (without reactivation of disease) for at least 6 months. To meet the criteria for “active” disease, patients were required to have new onset of uveitic disease occurrence at the time of recruitment (a new diagnosis or reactivation of existing disease), which was regardless of treatment status. Disease activity was determined by clinical symptoms, examination with slit lamp biomicroscopy and clinical imaging (optical coherence tomography, fundus autofluorescence, and fluorescein angiography). Patients who met the study inclusion criteria and who were clinically assessed to have active disease (i.e., anterior chamber cells ≥0.5+, vitreous cells ≥0.5+, inflammatory cystoid macular edema, optic disk edema, active vasculitis, or new/active chorio-retinal lesions) were considered eligible as “active” patients in the study. As controls, 10 age-matched subjects with no history of uveitis, systemic inflammatory disease or known infectious disease were enrolled onto the study.

A 30 mL sample of heparinized peripheral venous blood was obtained from all subjects. In the active cohort, samples were obtained before starting/modifying the immunosuppressive treatment regime. Additional peripheral venous blood samples were prospectively obtained from four active patients at 2, 6, and 12 months after starting/changing treatment.

This study was carried out in accordance with the recommendations of the UK National Research Ethics Service—London Harrow Committee and the Moorfields Eye Hospital National Health Service Foundation Trust, Department of Research and Development (13/LO/1653; 16039). All subjects gave written informed consent in accordance with the Declaration of Helsinki.

### Peripheral Blood Mononuclear Cells (PBMC) Isolation, Cell Culture, and Cryopreservation

Peripheral blood mononuclear cells were obtained from fresh heparinized blood (≤4 h following venupuncture) by Histopaque density gradient centrifugation (Histopaque-1077, Sigma-Aldrich, Gillingham, UK) and washed twice with RPMI 1640 with 10% human AB Serum (Sigma-Aldrich). 10 × 10^6^ PBMC from each sample were stained for multi-color flow cytometry.

For experimental cell culture conditions requiring stimulated cells, freshly isolated PBMC were cultured in X-VIVO 15 medium with l-glutamine, gentamicin, and phenol red (Lonza, Cambridge, UK) for 5 days, with the addition of 5 µg/mL soluble anti-CD3 (HIT3a) NA/LE, 1 µg/mL anti-CD28 NA/LE (BD Biosciences), and 50 IU IL-2 IS/mL (Miltenyi Biotec, Woking, UK) to the culture media, unless otherwise specified.

The remaining PBMC were cryopreserved in human AB serum with 10% Hybri-Max Sterile-filtered DMSO (Sigma-Aldrich) at −20°C for 1 h before transfer to −70°C and then to liquid nitrogen for longer term storage, for subsequent DNA isolation and methylation analysis.

### Antibodies

For multi-color immunophenotyping by flow cytometry, the following Abs were used: RORγt-Alexa Fluor 488, T-bet-PerCP-Cy5.5, CD3-Vioblue, CD25-Brilliant Violet 711, CD4-Brilliant Ultraviolet 395, FoxP3-Alexa Fluor 647, TIGIT-PE, and Near Infrared live/dead fixable cell stain. For intracellular cytokine staining by flow cytometry, the following Abs were used: CD3-Vioblue, CD4-Brilliant Ultraviolet 395, IL-10-PE-CF594, IFNγ-BV605, IL-17A PE-Vio-770, and Near Infrared live/dead fixable cell stain. For live cell sorting, the following Abs were used: CD4-PerCP-Cy5.5, CD127-Brilliant Violet 605, CD3-Brilliant Violet 785, CD25-PE-Dazzle 594, CD14-APC-Fire, and SYTOX blue dead cell stain. Abs were provided by BioLegend (London, UK), BD Biosciences (Oxford, UK), eBiosciences (Hatfield, UK), Miltenyi Biotec, and ThermoFisher Scientific (Dartford, UK).

### Treg and Th1/Th17 Immunophenotyping by Flow Cytometry

The peripheral blood levels of the following cell subsets were determined by flow cytometry: CD25^+^FoxP3^+^ Treg, CD25^+^FoxP3^+^TIGIT^+^ Treg, T-bet^+^ Th1, RORγt^+^ Th17, T-bet^+^RORγt^+^ Th1/17, Foxp3^+^T-bet^+^ Treg, and Foxp3^+^RORγt^+^ Treg in the CD3^+^CD4^+^ subset. Single-cell suspensions were washed in PBS (Sigma-Aldrich) and stained with directly conjugated Abs for cell surface molecules (CD3, CD4, CD25, TIGIT), then stained with Zombie NIR (BioLegend) fixable dead cell stain, then fixed and permeabilized with a transcription factor fixation/permeabilization kit (eBiosciences), and subsequently stained using directed conjugated Abs recognizing transcription factors (FoxP3, RORγt, and T-bet). Subject-specific fluorescence-minus-one (FMO) controls for CD25, TIGIT, FoxP3, RORγt, and T-bet were included in each experiment.

**Figure 1:**
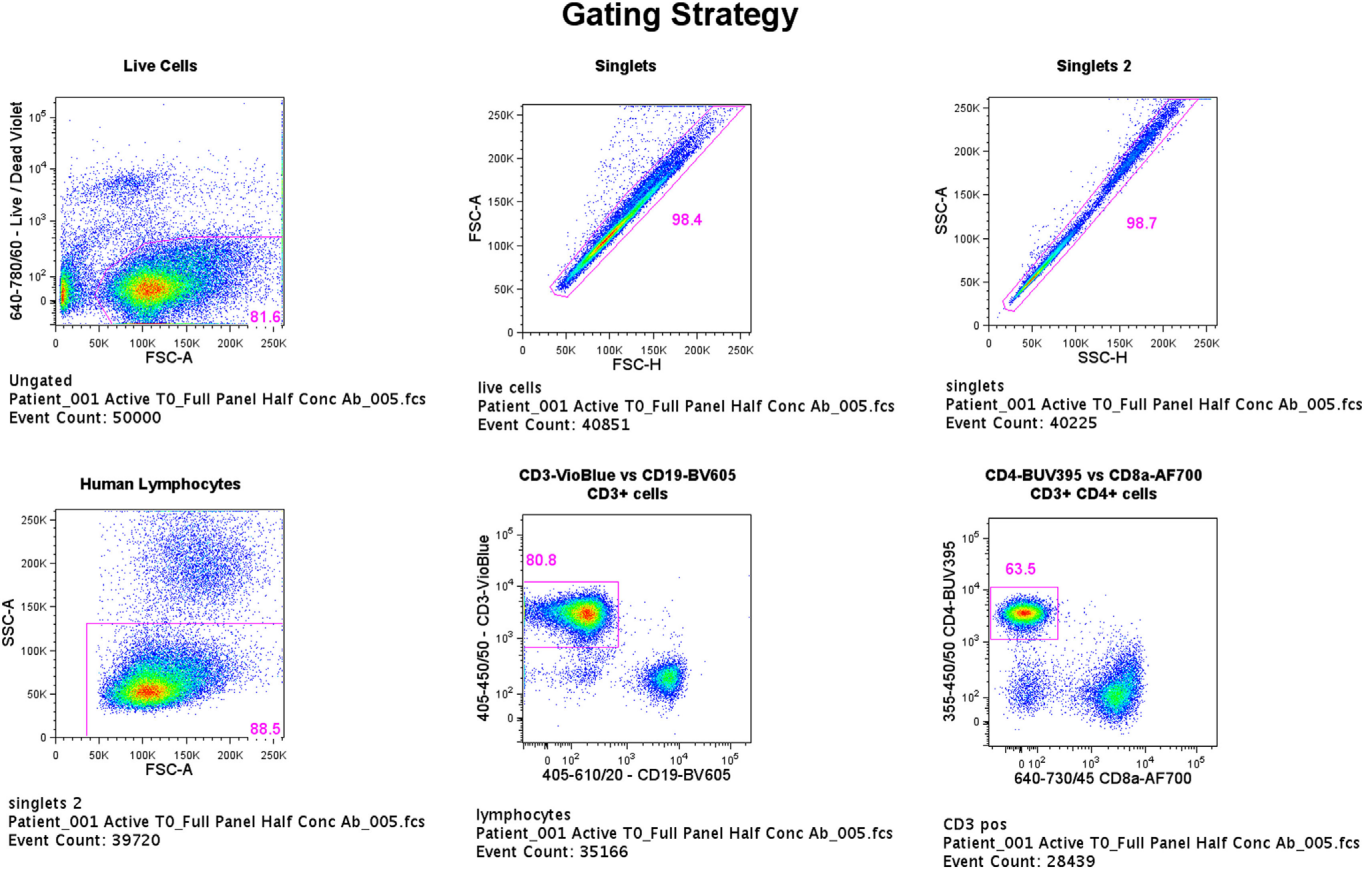
Flow cytometry gating strategy. Flow cytometric data were acquired using an LSRFortessa (BD Biosciences, San Jose, CA, USA) and BD FACSDiva version 6.1.3 software with a total of 50,000 events being recorded for each sample through a live, single-cell CD3^+^CD4^+^ gate. Viable cells were identified by low uptake of the fixable dead cell stain. Single-stained OneComp and UltraComp beads (eBiosciences) were used to generate compensation matrices. Fluorescence-minus-one controls were used to identify gating boundaries where cell populations were ill defined.

Flow cytometric data were acquired using an LSRFortessa (BD Biosciences, San Jose, CA, USA) and BD FACSDiva version 6.1.3 software with a total of 50,000 events being recorded for each sample through a live, single-cell CD3^+^CD4^+^ gate (gating strategy shown in Figure 1). Viable cells were identified by low uptake of the fixable dead cell stain. Single-stained OneComp and UltraComp beads (eBiosciences) were used to generate compensation matrices. FMO controls were used to identify gating boundaries where cell populations were ill-defined. Analysis of flow cytometry data was performed using FlowJo version 10 software (TreeStar Inc., Ashland, OR, USA).

### Cytokine Analysis

Whole blood samples were centrifuged following collection to obtain serum supernatant samples, which were stored in 1.5 mL aliquots at −70°C for subsequent multiplex cytokine analysis. Cryopreserved subject serum cytokine samples were thawed and immediately analyzed for cytokines IL-10, IFNγ, IL-17A, and IL-22 by multiplex bead arrays (R&D Systems) using a MAGPIX Luminex system (Luminex, TX, USA). The data were analyzed using xPONENT software (Luminex). Acidified serum aliquots from the same subject samples were analyzed for TGF-β by ELISA (R&D Systems) using the optical density setting of the Modulus II Microplate Multimode Reader (Promega Corporation). Minimum levels of detection based on standard curves for each array were as follows: IL-10 (7.30 pg/mL), IFNγ (17.30 pg/mL), IL-17A (2.65 pg/mL), IL-22 (6.10 pg/mL), and TGF-β (7.4 pg/mL).

For intracellular cytokine staining, freshly isolated PBMC from a patient with active disease (Patient 1) at 2 and 6 months after baseline were stimulated with Dynabeads Human T-Activator CD3/CD28 (ThermoFisher Scientific) for 24 h or a cell stimulation cocktail (containing PMA and ionomycin) 1:500 for 6 h as a positive control, with the addition of protein transport inhibitor (containing Brefeldin A) to the culture medium for the last 4 h. Staining and flow cytometric analyses were as described earlier, with the following modifications: directly conjugated Abs for cell surface molecules (CD3 and CD4); fixation and permeabilization with an intracellular cytokine fixation/permeabilization kit (BD Biosciences); and directly conjugated Abs recognizing intracellular cytokines (IL-10, IFNγ, and IL-17A). Subject-specific FMO controls for IL-10, IFNγ, and IL-17A were included in each experiment.

### Cell Sorting and T-Cell Proliferation Assay

**Figure 2:**
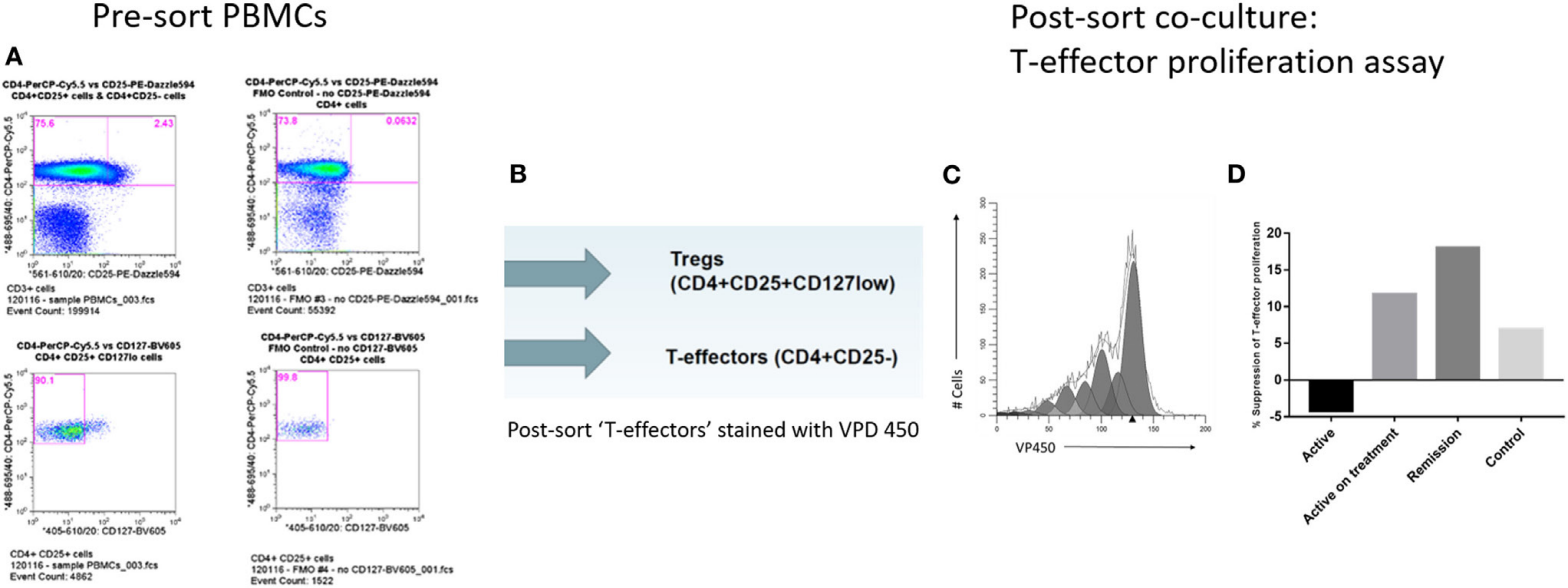
Cell sorting and T-regulatory cell (Treg) suppression assays. **(A)** Cell sorting strategy to isolate Treg (CD4^+^CD25^+^CD127^lo^) and T-effectors (CD4^+^CD25^−^) in CD3^+^ T-cell subset. **(B)** Post-cell sort staining of T-effectors with violet proliferation dye (VPD) 450 cell tracking dye. **(C)** Modfit modeling of *in vitro* T-cell proliferation index using VPD 450. **(D)** Comparison of % suppression of T-cell proliferation by Treg isolated from the different subject groups.

Freshly isolated PBMCs from subject samples in each group were stained with CD3, CD4, CD25, CD127, and CD14 cell surface Abs. CD3^+^CD4^+^CD25^+^CD127^lo^, CD3^+^CD4^+^CD25^−^, and CD14^+^ populations representing Treg, putative T-effectors, and monocyte (MC) subsets, respectively, were isolated directly from the stained PBMC by means of high speed Influx flow cytometric cell sorting (BD Biosciences, San Jose, CA, USA) (gating strategy shown in Figure 2A).

CD3^+^CD4^+^CD25^−^ T-cells were washed and resuspended at 10 × 10^6^/mL in sterile PBS (Sigma-Aldrich) containing 1 µL of 1 mM violet proliferation dye (VPD) 450 stock solution (BD Biosciences) for each 1 mL of cell suspension for a final VPD450 concentration of 1 µM, according to the manufacturer’s instructions (Figure 2B). The cells were stained by incubating the dye-cell suspension in a 37°C water bath for 10 min. The reaction was quenched by adding 9× the original volume of PBS to the cells, followed by centrifugation, discarding the supernatant, and resuspending the cells in 10 mL of RPMI 1640 medium with 10% FBS before washing again.

The *in vitro* capacity of the Treg to suppress the proliferation of VPD450-labeled CD3^+^CD4^+^CD25^−^ responding T-cells (Tresp) was assessed in 96-well plates (1 × 10^6^ per well density) in a classical 5-day coculture assay, as follows: VPD450-labeled CD3^+^CD4^+^CD25^−^ Tresp were cocultured with sorted CD14^+^ MCs at 1:1 ratio and sorted CD3^+^CD4^+^CD25^+^CD127^lo^ Tregs were cocultured with Tresp and MC at a 1:3:3 ratio. Cell culture conditions were as previously described. Data were acquired by flow cytometry and analyzed using the cell tracking function of Modfit LT modeling software (Verity Software House, ME, USA) to generate a statistic termed proliferation index (PI) (Figure 2C). Percentage (%) suppression was determined for each subject sample as follows, adapted from previously described methods for conducting suppression assays from small numbers of isolated T-cells (15, 40):

$$\%\text{ suppression}=\frac{\left(\text{Tresp}+\text{MC})-(\text{Tresp}+\text{MC}+\text{Treg}\right)}{\left(\text{Tresp}+\text{MC}\right)}\times 100.$$

### DNA Isolation and Methylation Analysis

**Figure 3:**
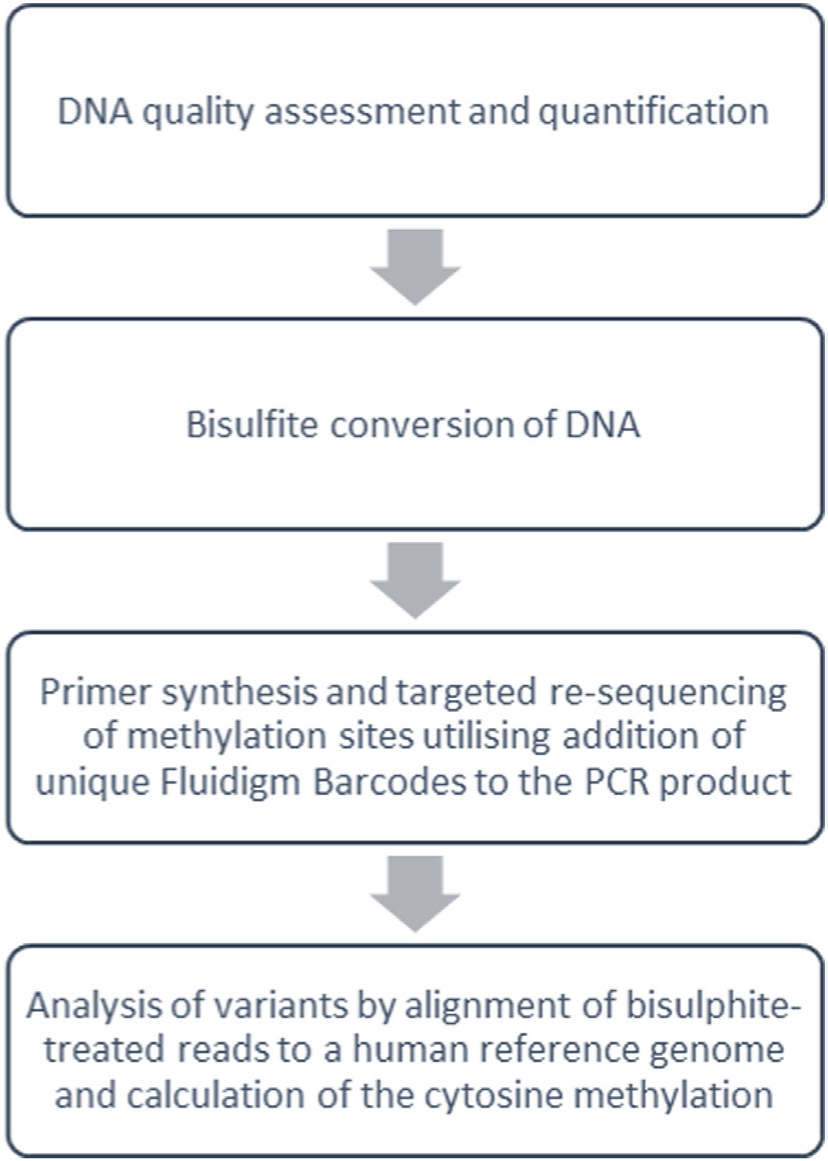
Overview of targeted DNA methylation analysis methods. Summary work-flow of bisulfite Amplicon Sequencing using a next-generation sequencing approach with the Illumina sequencing platform (Fluidigm, CA, USA).

Genomic DNA extraction was performed directly on samples of thawed cryopreserved PBMC from each subject group using the DNeasy Blood and Tissue Kit (Qiagen, Manchester, UK). The samples were then analyzed at five DNA sites of cytosine (CpG) methylation (FOXP3 TSDR, FOXP3 promoter, TBX21, RORC2, and TIGIT) with bisulfite Amplicon Sequencing using an NGS approach on the Illumina sequencing platform (Fluidigm, CA, USA) (Figure 3; bisulfite Amplicon target sites are shown in Figure S1 in Supplementary Material).

Before DNA bisulfite treatment, the integrity of the DNA was assessed using the Agilent 2100 Bioanalyser Tapestation (Agilent Technologies, Waldbronn, Germany), and the DNA concentration was measured using Qubit Fluorometric Quantification (ThermoFisher Scientific). 500 ng of normalized DNA was bisulfite converted using the EZ DNA Methylation kit (Cambridge BioSciences, Cambridge). The incubation conditions recommended by the manufacturers guide [95°C for 30 s, 50°C for 60 min] × 16 cycles, 4°C hold were used for the conversion reaction. The yield of bisulfite converted DNA was estimated using the RNA-40 setting on the NanoDrop 8000 Spectrophotometer V2.0 (ThermoScientific, USA). Primers for the target sites were designed using Pyromark Assay Design 2.0 software (Qiagen) and synthesized with Fluidigm Universal CS1 and CS2 tags (Fluidigm, San Francisco, USA) to allow for the construction of DNA libraries using Fluidigm proprietary indexes (see Table S2 in Supplementary Material for primer sequences *Fluidigm CS1 and CS2 tags are highlighted in bold*). MyTaq™ HS DNA Polymerase (Bioline, USA) was used to optimize the assays and amplify the DNAs. The PCR products were checked for amplification on an Agilent 2100 Bioanalyser D1K Tape (Agilent Technologies, Waldbronn, Germany) after which unique Fluidigm Barcodes (Table S3 in Supplementary Material) were added to the PCR product under the following PCR cycling conditions: 95°C for 10 min (95°C for 15 s, 60°C for 30 s, and 72°C for 1 min) cycle 14 times; 4°C hold. To check that the barcodes had been successfully incorporated onto the PCR products, the barcoded PCR product was run on an Agilent D1K tape. If the barcodes had attached, then a shift of ~60 bp was observed between the PCR product and the barcoded products. Equal volumes of barcoded products were pooled and primer dimer was removed using Agencourt Ampure XP beads (Beckman Coulter Life Sciences) at a volume of 1:1. The pool was quantified using High Sensitivity Qubit Reagents (Qiagen), and the library size was measured using the Agilent 2100 High Sensitivity D1K (Agilent Technologies, Waldbronn, Germany) to calculate the molarity of the library before sequencing. 150-bp Paired-end sequencing was performed using the Miseq Illumina sequencer (Illumina, Inc.) to a total of 400,000 reads passing filter per sample. Bismark (41) was used to align bisulfite-treated reads to a human reference genome and calculate the cytosine methylation calls at the target sites. Biostatistical analysis generated beta (%) methylation values as a ratio of methylated to unmethylated DNA for each CpG site of interest.

### Statistical Analysis

Statistical analysis of final datasets was performed with SPSS version 24 (IBM Software) and GraphPad Prism version 7 (GraphPad Software). Differences between groups were determined by the Kruskal–Wallis test with *post hoc* Bonferroni correction for multiple comparisons. Bivariate correlations between immunological variables were calculated using Spearman’s test. Relationships between selected variables, which had clinically relevant associations, were modeled using multiple linear regression and logistic regression, using “stepwise” or “enter” variable entry, respectively. Where possible, variables with a non-normal distribution were transformed to a normal distribution, using a log transformation, to include in the multiple regression model. All significance tests were two-tailed. *P*-values < 0.05 were considered significant. Results are expressed as frequency *n* (%) or median (IQR), unless otherwise stated.

## Results

### Subject Characteristics

**Figure 4:**
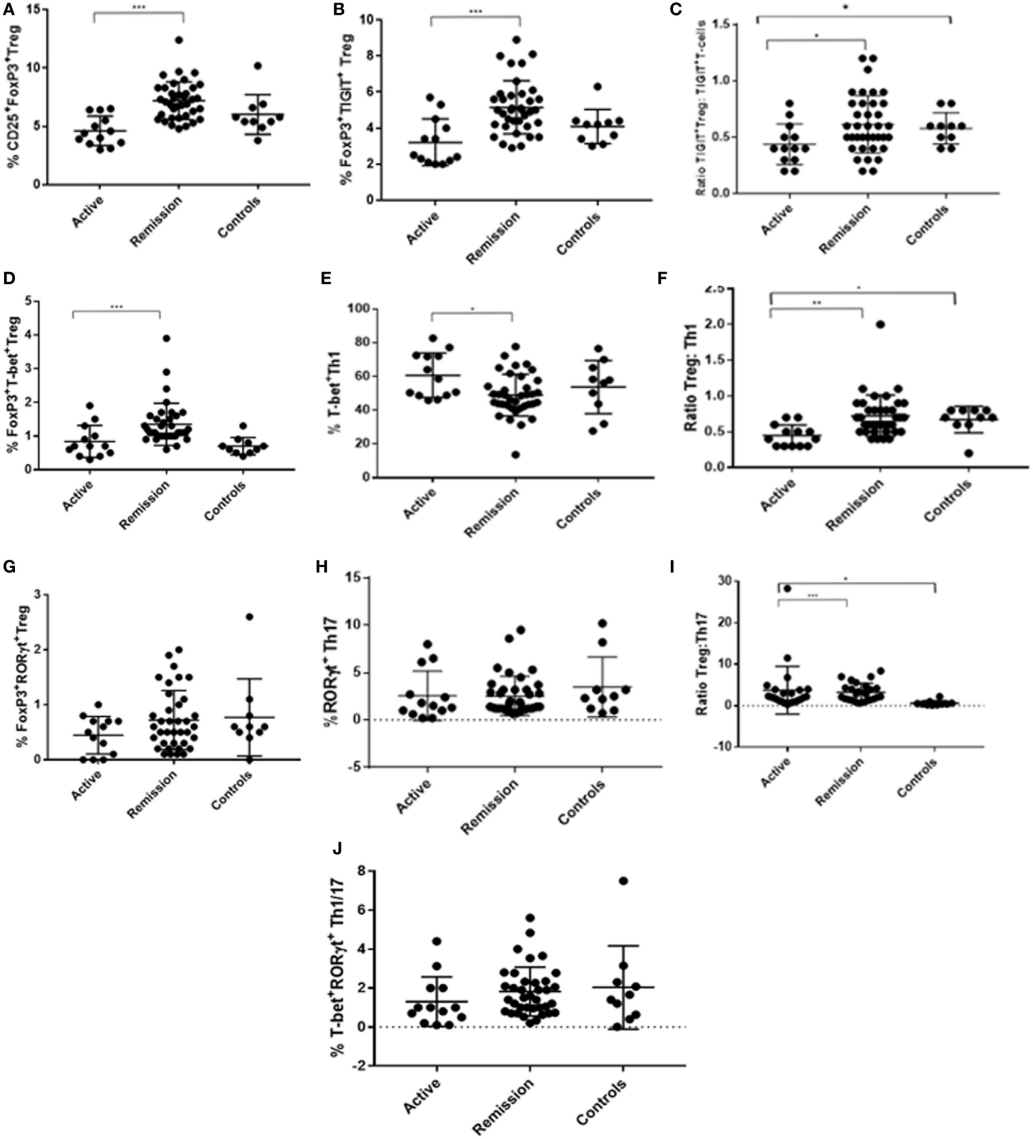
Dot plots showing flow cytometry analysis results (% frequency or ratio) of peripheral blood levels of T-regulatory cell (Treg), Th1, and Th17 subtypes in CD3^+^CD4^+^ T-cell compartment, with comparisons across the three subject groups (active, remission, and controls). **(A)** % CD25^+^FoxP3^+^ Treg. **(B)** % FoxP3^+^TIGIT^+^ Treg. **(C)** Ratio of FoxP3^+^TIGIT^+^ Treg to TIGIT^+^FoxP3^−^ T-cells. **(D)** % FoxP3^+^T-bet^+^ Treg. **(E)** % T-bet^+^ Th1. **(F)** Ratio of Treg to Th1. **(G)** % FoxP3^+^RORγt^+^ Treg. **(H)** % RORγt^+^ Th17. **(I)** Ratio of Treg to Th17. **(J)** % T-bet^+^RORγt^+^ “double positive” Th1/Th17.

A total of 50 uveitis patients and 10 control subjects were recruited to the study (Table 1). Of the 50 patients recruited, 37 were in clinical remission and 13 had active disease at the time of recruitment. 22 (59%) of the patients in the clinical remission group received previous therapy with corticosteroids only, the remaining patients received additional second-line oral immunosuppressive treatment (Table 2). Of the active patients, who all had intermediate uveitis, posterior uveitis or panuveitis, 11 (85%) had cells and/or haze in the vitreous, 7 (54%) had cells in anterior chamber, 6 (46%) had macular edema, 3 (23%) had optic disk edema, two (15%) had vasculitis, and 1 (8%) had chorio-retinal lesions at baseline. Six (46%) of these patients with active disease were on systemic immunosuppression at the time of recruitment. A subgroup analysis comparing the active patients on therapy and active patients not on therapy at baseline did not reveal any differences between the groups. Four of the 13 active patients underwent additional immunophenotyping at 2, 6, and 12 months after starting or changing treatment. The immunological marker levels and comparison across the three subject groups (active, remission, and controls) with summary *P*-values are shown in Table 3 and Figure 4, with the *post hoc* pairwise comparisons and adjusted *P*-values shown in Table S1 in Supplementary Material.

**Table 1:** Subject demographics and clinical characteristics.

Characteristics	Control (*n* = 10)	Active (*n* = 13)	Quiescent (*n* = 37)
Median age in years (IQR)	**30** (29–33)	**41** (27–46)	**47** (35–55)
Female sex—no. (%)	**6** (60)	**6** (46)	**19** (51)
**Race or ethnic group, no. (%)**		
White/European	**6** (60)	**6** (46)	**26** (70)
Asian/Middle Eastern	**4** (40)	**3** (23)	**10** (27)
Black/Afro-Carribean	0	**4** (31)	**1** (3)
**Uveitis SUN classification**		
Intermediate	–	**6** (46)	**16** (43)
Posterior	–	**1** (8)	**6** (16)
Panuveitis	–	**6** (46)	**15** (41)
Bilateral disease, no. (%)	–	**10** (77)	**30** (81)
Median visual acuity (log MAR)	–	**0.2** (0.1–0.4)	**0.2** (0.1–0.5)
Visually significant cataract (%)	–	**2** (15)	**9** (24)
Previous cataract surgery	–	**2** (15)	**15** (41)
Median disease duration since diagnosis, months (IQR)	–	**33** (7–119)	**147** (50–249)

*Median values are in bold font*.

**Table 2:** Subject immunosuppressive treatment characteristics.

	Control (*n* = 10)	Active (*n* = 13)	Quiescent (*n* = 37)
On oral immunosuppressive treatment at baseline, no. (%)	–	**6** (46)	**0** (0)
Previous oral immunosuppression received, no. (%)		**10** (77)	**37** (100)
Prednisolone only		**4** (40)	**22** (59)
Additional second-line immunosuppression		**6** (60)	**15** (41)
Mycophenolate mofetil	–	**3** (23)	**5** (14)
Ciclosporin	–	**0** (0)	**7** (20)
Azathioprine	–	**1** (8)	**2** (5)
Methotrexate	–	**0** (0)	**1** (3)
Biologicals	–	**0** (0)	**1** (3)
Median duration of previous oral immunosuppression, months (IQR)	–	**6** (4–12)	**24** (7–72)

*Median values are in bold font*.

**Table 3:** Comparison of immunological markers across the three subject groups.

Immunological marker	Subject cohort			3-Way comparison
	
	Control	Active	Remission	Summary *p*-value
**T-cell subsets (%) and ratios**				
CD25^+^FoxP3^+^ T-regulatory cell (Treg)	**5.6** (5.3–6.7)	**4.4** (3.6–6.0)	**7.1** (5.8–8.3)	0.000***
CD25^+^TIGIT^+^FoxP3^+^ Treg	**4.2** (3.3–4.4)	**2.5** (2.1–4.3)	**5.0** (4.2–5.9)	0.000***
TIGIT^+^FoxP3^+^:TIGIT^+^FoxP3^−^	**0.6** (0.5–0.7)	**0.4** (0.2–0.6)	**0.6** (0.5–0.8)	0.049*
TIGIT^+^FoxP3^+^CD25^+^:TIGIT^+^FoxP3^−^	**0.5** (0.4–0.6)	**0.3** (0.1–0.4)	**0.7** (0.5–0.9)	0.003**
Th1 (T-bet^+^)	**57.1** (40.8–66.4)	**58.6** (48.8–72.2)	**48.5** (42.7–55.9)	0.025*
Th17 (RORγt^+^)	**2.4** (1.2–4.5)	**1.5** (0.8–4.4)	**1.7** (1.2–3.2)	0.564
Treg:Th1	**0.7** (0.6–0.8)	**0.4** (0.3–0.6)	**0.7** (0.5–0.9)	0.000***
Treg:Th17	**0.4** (0.2–0.4)	**2.0** (0.8–3.5)	**2.8** (1.5–4.3)	0.001**
T-bet^+^FoxP3^+^	**0.7** (0.5–0.8)	**0.7** (0.5–1.2)	**1.1** (1.0–1.6)	0.000***
RORγt^+^FoxP3^+^	**0.6** (0.5–0.9)	**0.5** (0.1–0.7)	**0.6** (0.3–1.1)	0.363
RORγt^+^T-bet^+^	**1.5** (0.6–2.5)	**1.0** (0.4–2.0)	**1.5** (0.9–2.3)	0.183

**Serum cytokine levels (pg/mL)**				
IL-10	**24.2** (22.8–25.4)	**11.6** (9.8–11.8)	**21.5** (20.0–22.8)	0.000***
Transforming growth factor β	**56.9** (38.9–85.4)	**62.7** (48.7–96.5)	**161.1** (122.5–285.2)	0.001**
IFN-γ	**208.4** (197.3–214.4)	**408.8** (404.8–418.8)	**297.8** (296.1–299.5)	0.000***
IL-17	**13.9** (13.2–14.0)	**14.8** (13.9–15.9)	**13.0** (11.9–13.4)	0.003**
IL-22	**19.9** (17.3–20.2)	**20.6** (20.3–22.0)	**18.3** (17.1–18.8)	0.002**

**CpG site methylation levels (%)**				
FOXP3 promoter	**69** (52–75)	**72** (66–77)	**53** (46–68)	0.037*
FOXP3 Treg-specific demethylated region	**69** (61–71)	**76** (68–83)	**63** (59–67)	0.005**
TIGIT	**54** (47–55)	**59** (57–64)	**48** (46–53)	0.004**
TBX21/T-BET	**0.7** (0.6–0.8)	**0.4** (0.3–0.5)	**0.7** (0.5–0.8)	0.122
RORC2/RORγT	**55** (52–61)	**46** (44–51)	**55** (51–58)	0.006*

*Median values are in bold font*.

*Significant at the 5% level.

**Significant at the 1% level.

***Significant at the 0.1% level.

### Clinical Remission of Uveitis Is Associated With Higher Levels of Treg Polarized Toward T-bet and TIGIT Compared With Active Disease

**Figure 5:**
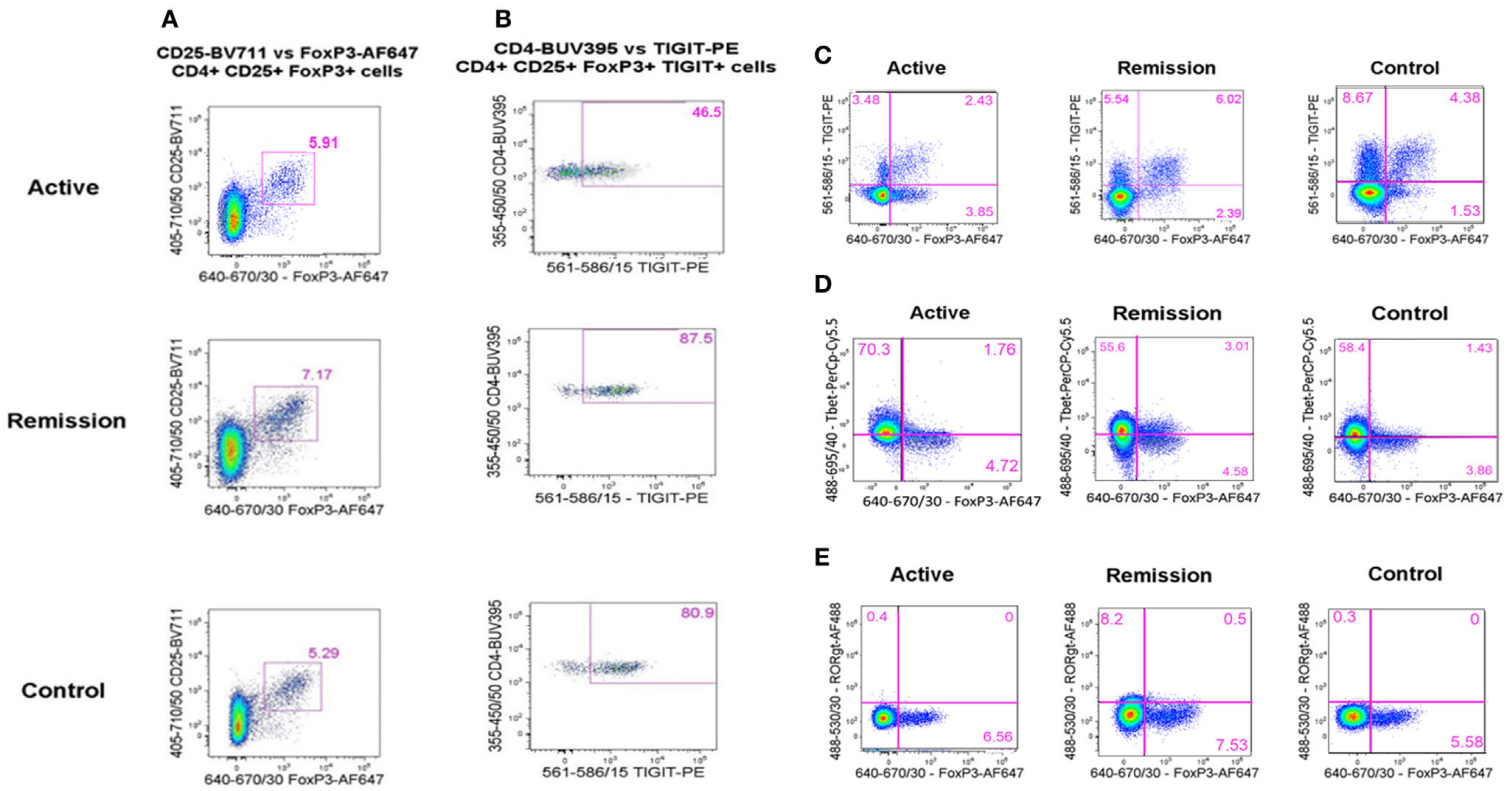
Flow cytometry analysis plots showing representative examples of peripheral blood levels of T-regulatory cell (Treg) subtypes in the CD3^+^CD4^+^ T-cell compartment, across the three subject groups (active, remission, and controls). **(A)** % CD25^+^FoxP3^+^ Treg. **(B)** % TIGIT^+^ Treg in the (above) CD25^+^FoxP3^+^ Treg subset. **(C)** % FoxP3^+^TIGIT^+^ Treg (upper right quadrant) and TIGIT^+^FoxP3^−^ T-cells (upper left quadrant). **(D)** % FoxP3^+^T-bet^+^ Treg (upper right quadrant). **(E)** % FoxP3^+^RORγt^+^ Treg (upper right quadrant).

**Figure 6:**
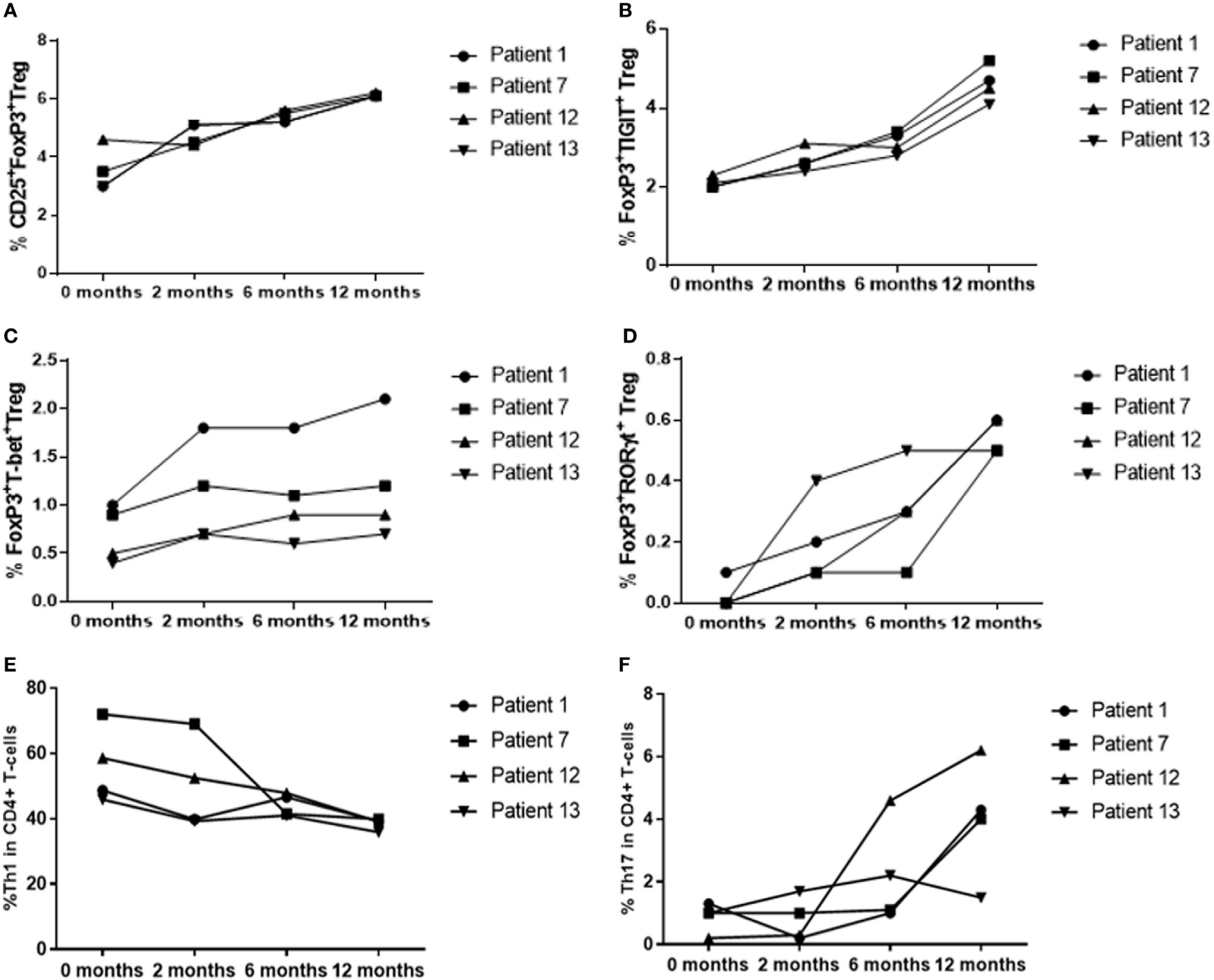
T-regulatory cell (Treg), Th1 and Th17 % levels in patients with active disease at baseline, as disease resolved over 12 months of treatment with oral immunosuppression. **(A)** CD25^+^FoxP3^+^ Treg. **(B)** CD25^+^FoxP3^+^TIGIT^+^ Treg. **(C)** FoxP3^+^T-bet^+^ Treg. **(D)** FoxP3^+^RORγt^+^ Treg. **(E)** CD4^+^T-bet^+^ Th1. **(F)** CD4^+^RORγt^+^ Th17.

To determine whether there was a difference in peripheral blood Treg levels between control, active and quiescent subjects in disease remission, their PBMC were analyzed by flow cytometry for levels of CD25^+^FoxP3^+^ Treg, FoxP3^+^TIGIT^+^ Treg, FoxP3^−^TIGIT^+^ T-cells, and FoxP3^+^T-bet^+^ Treg in the CD3^+^CD4^+^ lymphocyte subset (Figure 5). Highly statistically significant differences were found between the active and remission groups in the levels of Treg (4.4 ± 1.2 vs 7.1 ± 1.3, *P* = 0.000), TIGIT^+^ Treg (2.5 ± 1.3 vs 5.0 ± 0.9, *P* = 0.000) and T-bet^+^ Treg (0.7 ± 0.4 vs 1.1 ± 0.3, *P* = 0.000) (Figure 4). CD25^+^FoxP3^+^ Treg, FoxP3^+^TIGIT^+^ Treg, and FoxP3^+^T-bet^+^ Treg were also observed to increase as disease resolved in patients with active uveitis (Figure 6). The ratio of CD4^+^CD25^+^FoxP3^+^TIGIT^+^ Treg to CD4^+^FOXP3^−^TIGIT^+^ T-cells was significantly higher in the remission group compared with the active group (0.7 ± 0.2 vs 0.3 ± 0.2, *P* = 0.003). T-bet^+^FoxP3^+^ cell levels positively correlated with TIGIT^+^FoxP3^+^ cell levels across the three subject groups (*r* = 0.431, *P* = 0.001). There were no statistically significant differences between Treg levels in control and active uveitis patient groups.

### FoxP3^+^RORγt^+^ Tregs Are Not Associated With Clinical Remission but May Have a Role in Clinical Resolution of Non-Infectious Uveitis

Peripheral blood levels were analyzed for CD3^+^CD4^+^FoxP3^+^RORγt^+^ Treg (Figure 5). Although the remission and control groups had higher median levels of FoxP3^+^RORγt^+^ Treg compared with the active group, these differences did not reach statistical significance (*P* = 0.363) (Figure 4). However, an overall increase in FoxP3^+^RORγt^+^ Treg levels was observed over 12 months in all four patients with active disease at baseline (Figure 6).

### Clinical Remission of Uveitis Is Associated With Overall Lower Levels of Th1 Transcription Factors and a Higher Ratio of Treg to Th1 Compared With Active Disease

To determine whether there was a difference in Th1 (T-bet transcription factor expression) levels and ratios of Treg to Th1 between control, active and quiescent subjects in disease remission, their PBMC were analyzed by flow cytometry for levels of CD25^+^FoxP3^+^ Treg (Figure 5) and T-bet^+^ T-cells in the CD3^+^CD4^+^ lymphocyte subset. Significantly lower levels of T-bet transcription factor levels (48.5 ± 6.8 vs 58.6 ± 11.7, *P* = 0.024) and higher ratios of Treg:Th1 (0.7 ± 0.2 vs 0.4 ± 0.1, *P* = 0.001) were found in remission patients compared with active patients (Figure 4). Active patients also had significant lower ratios of Treg:Th1 compared with control subjects (0.4 ± 0.1 vs 0.7 ± 0.1, *P* = 0.013) (Figure 4). Disease resolution in active patients over the course of 12 months appeared to be associated with an increase in levels of Treg and decrease in levels of Th1 (Figure 6).

### Clinical Remission of Uveitis Is Not Associated With Overall Lower Levels of Th17 Transcription Factors or a Higher Ratio of Treg to Th17 Compared With Active Disease

To determine whether there was a difference in Th17 (RORγt^+^ transcription factor expression) levels and Treg:Th17 ratios between control, active and quiescent subjects in disease remission, their PBMC were analyzed by flow cytometry for levels of CD25^+^FoxP3^+^ Treg (Figure 5) and RORγt^+^ T-cells in the CD3^+^CD4^+^ lymphocyte subset. No significant difference was found in Th17 levels between the three groups (*P* = 0.564), although it was noted that both active (2.0 ± 1.3) and remission (2.8 ± 1.4) patients had significantly higher Treg:Th17 ratios in comparison with control subjects (0.4 ± 0.3, *P* = 0.014; *P* = 0.000 respectively) (Figure 4). Increased Th17 levels in three out of four patients with active disease were noted as the disease clinically resolved over 12 months (Figure 6).

### Clinical Remission of Uveitis Is Not Associated With Decreased Levels of “Double Positive” Effector RORγt^+^T-bet^+^ T-Cells

Peripheral blood levels were analyzed for “double positive” RORγt^+^T-bet^+^ T-cells in the CD3^+^CD4^+^ subset. There was no significant difference in levels of RORγt^+^T-bet^+^ T-cells between the three groups (*P* = 0.183) (Figure 4).

### Clinical Remission of Uveitis Is Associated With Higher Serum Levels of TGF-β and IL-10 and Lower Serum Levels of IFNγ, IL-17A, and IL-22 Compared With Active Disease, and Serum TGF-β and IL-10 Levels Positively Correlate With Treg Levels

**Figure 7:**
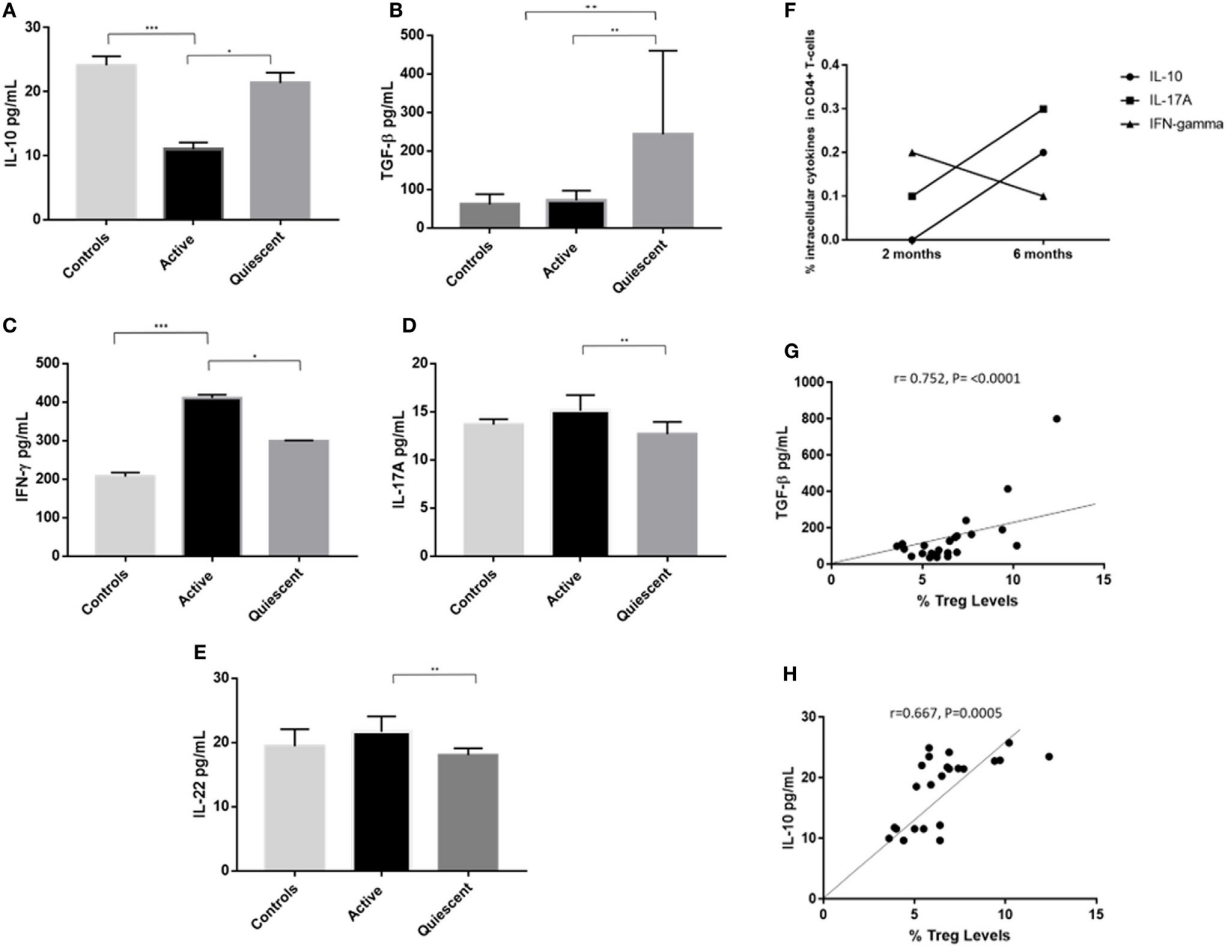
Serum cytokines levels (pg/mL) of IL-10, IFNγ, IL-17A, IL-22, and transforming growth factor β (TGF-β) in the three subject groups (active, remission, and controls), intracellular levels of IL-10, IFNγ, and IL-17A, and correlation of serum IL-10 and TGF-β levels with T-regulatory cell (Treg) levels. **(A)** Serum IL-10 levels. **(B)** Serum IFNγ levels. **(C)** Serum IL-17A levels. **(D)** Serum IL-22 levels. **(E)** Serum TGF-β levels. **(F)** Intracellular levels of IL-10, IFNγ, and IL-17A in the CD3^+^CD4^+^ compartment of T-cells in a patient with active disease at 2 and 6 months after starting immunosuppressive treatment, following 24 h *in vitro* culture with anti-CD3 and anti-CD28 bead stimulation. **(G)** Bivariate correlation of serum TGF-β (pg/mL) and % Treg frequency, across all subject groups. **(H)** Bivariate correlation of serum IL-10 levels (pg/mL) and % Treg frequency, across all subject groups.

Cytokine levels of IL-10, TGF-β, IFNγ, IL-17A, and IL-22 were assayed in serum from 10 remission patients, 8 active patients and 5 control subjects from the above groups (Figures 7A–E). IL-10 levels were significantly higher in serum from remission patients compared with active patients (21.5 ± 2.4 vs 11.6 ± 1.5, *P* = 0.011), and lower in active patients compared with controls (11.6 ± 1.5 vs 24.2 ± 2.3, *P* = 0.000). TGF-β levels were higher in remission patients than in active (161.1 ± 81.4 vs 62.7 ± 24.0, *P* = 0.007) and control (161.1 ± 1.5 vs 56.9 ± 24.0, *P* = 0.004) subjects. Clinical remission was also associated with significantly lower serum levels of IFNγ (*P* = 0.015), IL-17A (*P* = 0.002), and IL-22 (*P* = 0.001) compared with patients with active disease. Increased intracellular IL-10 and IL-17A and decreased intracellular IFNγ levels were found in CD4^+^ T-cells from active uveitis, 6 months after starting treatment, when the disease had clinically resolved (Figure 7F). Serum TGF-β (*r* = 0.752, *P* <0.0001) and IL-10 (*r* = 0.667, *P* = 0.0005) levels positively correlated with Treg levels across all subjects (Figures 7G,H).

### Clinical Remission of Uveitis Is Associated With Treg Which Demonstrate a High Capacity to Suppress Proliferating T-Effectors

Cell-sorted populations of Treg, putative T-effectors and macrophages were set up in *in vitro* culture and the T-cell PI was modeled by Modfit software, which was used to calculate% suppression by Treg, as described earlier. Treg from active uveitis without treatment failed to suppress proliferation of T-cells *in vitro* (Figure 2D). By contrast, Treg from active uveitis treated with oral immunosuppression, Treg from control subjects and Treg from uveitis in clinical remission all showed *in vitro* functional suppression, with the highest suppressive capacity demonstrated by Treg from uveitis in clinical remission (Figure 2D). Data are shown from the following subjects: Patients 1 and 50 (in active disease group), Patient 49 (in clinical remission group), and Volunteer 2 (in control group). Patient 50 had active disease and had not yet started oral immunosuppression, whereas Patient 1 had a diagnosis of active disease at baseline and had received 6 months of oral immunosuppression.

### Lower Levels of Methylation at Key Epigenetic CpG Sites Determining Treg Function in PBMC Are Associated With Clinical Remission but Are Not Consistently Observed in Clinical Resolution of Uveitis

**Figure 8:**
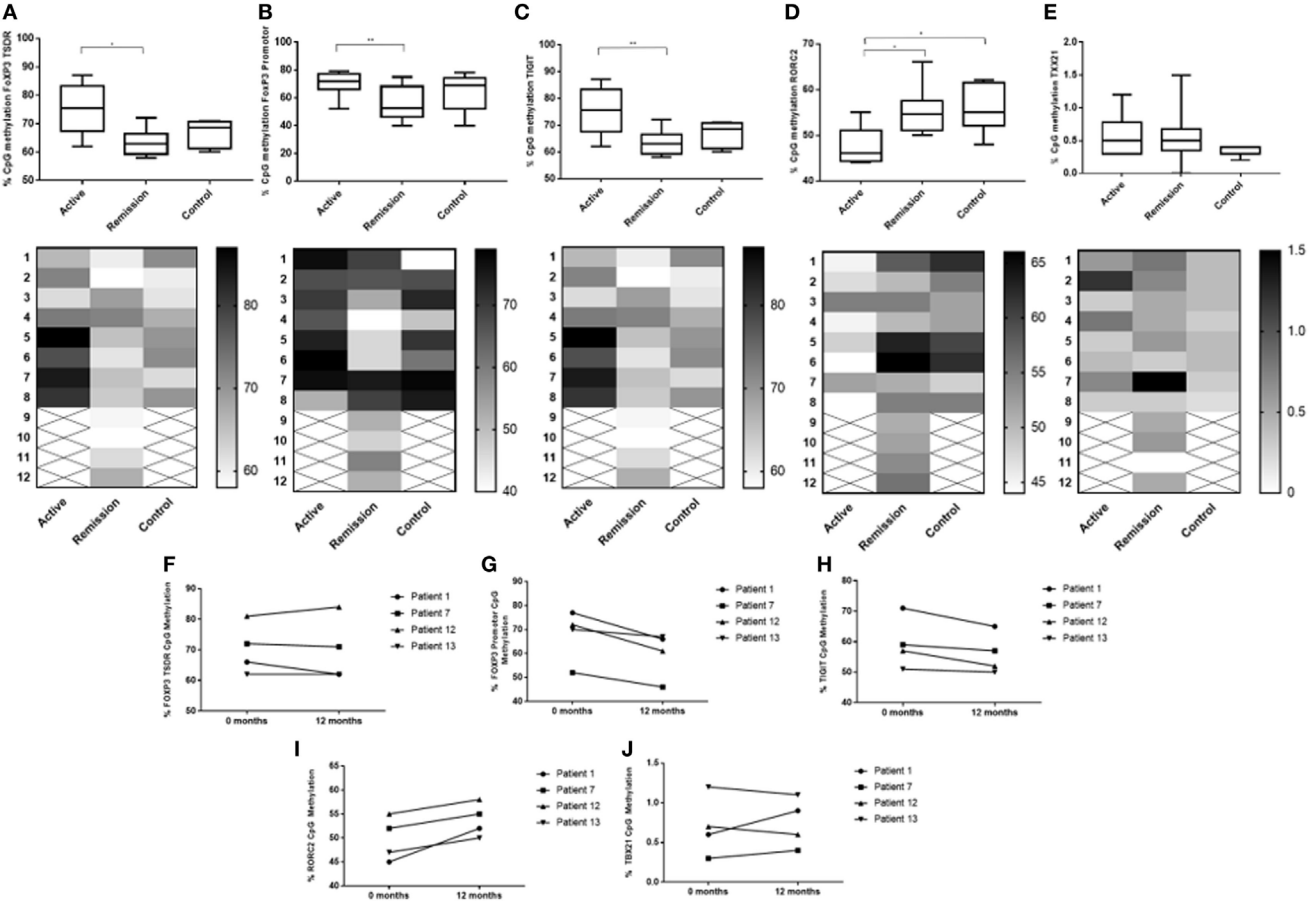
Comparison of % DNA CpG site methylation levels between the three subject groups **(A–E)** and change in % methylation within individual patients diagnosed with active disease at 0 month (commenced on oral treatment) and at 12 months, when disease had resolved **(F–J)**. **(A)** Between subject % methylation at FOXP3 Treg-specific demethylated region (TSDR) (49117116). **(B)** Between subject % methylation at FOXP3 promoter (49121204). **(C)** Between subject % methylation at TIGIT (114012658). **(D)** Between subject % methylation at RORC2/RORγT (151798858). **(E)** Between subject % methylation at TBX21/T-BET (45810951). **(F)** Change in individual % methylation at FOXP3 TSDR (49117116). **(G)** Change in individual % methylation at FOXP3 promoter (49121204). **(H)** Change in individual % methylation at TIGIT (114012658). **(I)** Change in individual % methylation at RORC2/RORγT (151798858). **(J)** Change in individual % methylation at TBX21/T-BET (45810951).

To determine whether there was a difference in methylation at epigenetic CpG sites associated with Treg function (FoxP3 and TIGIT), bisulfite Amplicon Sequencing using an NGS approach was performed using cryopreserved PBMC from 8 active patients, 8 control subjects and 12 remission patients. Cryopreserved served samples, at 12 months following treatment, were also obtained from four of the eight active patients. The sample consisted of 17 female subjects (61%) and 11 male subjects (39%). Following biostatistical analysis of the epigenetic percentage (%) methylation data, a single CpG site demonstrating a high level of differential methylation between samples was selected for statistical comparison between the three groups at that site, as follows: FoxP3 TSDR (49117116), FoxP3 promoter (49121204), and TIGIT (114012658) (Figure 8). The median CpG site% methylation levels of the FoxP3 TSDR (48 ± 3.6 vs 59 ± 7.4, *P* = 0.003) (Figure 8A), FoxP3 promoter (53 ± 11 vs 72 ± 5.3, *P* = 0.036) (Figure 8B), and TIGIT (48 ± 3.3 vs 59 ± 3.3, *P* = 0.003) (Figure 8C) CpG sites were significantly lower in patients in clinical remission compared with active patients. When comparing% CpG site methylation within individual patients with active disease at 0 month and resolved disease at 12 months, a decrease in methylation at the FoxP3 promoter was observed over time whereas the methylation of FoxP3 TSDR and TIGIT appeared more stable (Figures 8F–H).

### Increased Levels RORC Methylation May Be a Biomarker of Disease Response to Treatment

To determine whether there was a difference in methylation at epigenetic CpG sites associated with the T-effector transcription expression levels (RORγt and T-bet) assessed by flow cytometry in this study, bisulfite Amplicon Sequencing using an NGS approach was performed using cryopreserved PBMC as described earlier. Following biostatistical analysis of the epigenetic percentage (%) methylation data, a single CpG site demonstrating a high level of differential methylation between samples was selected for statistical comparison between the three groups at that site, as follows: RORC2/RORγT (151798858) and TBX21/T-BET (45810951) (Figure 8). The median CpG site% methylation level of RORC2 was higher in patients in clinical remission (55 ± 3.3 vs 46 ± 3.4, *P* = 0.016) and in control subjects compared with active (55 ± 4.3 vs 46 ± 3.4, *P* = 0.014) patients (Figure 8D). Within the active patient cohort, higher RORC% CpG methylation levels were observed at 12 months compared with 0 month after starting/changing immunosuppressive treatment (Figures 8I,J). TBX21 CpG sites had overall low levels of methylation compared with the other T-cell-associated epigenetic methylation sites and no significant difference in the median% methylation levels was found between the three cohorts (Figure 8E).

### Previous Duration of Oral Immunosuppressive Treatment Is a Significant Predictor of Methylation at FoxP3 TSDR

**Figure 9:**
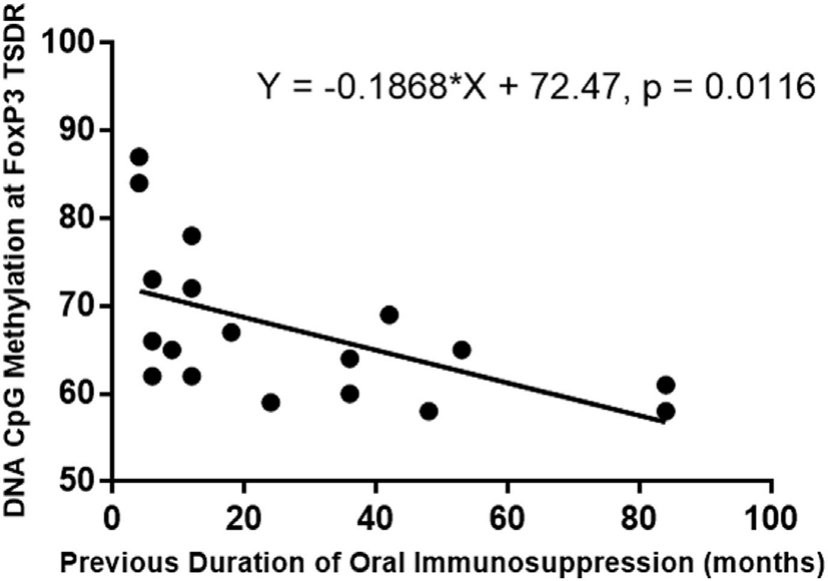
Simple linear regression of the duration of the previous oral immunosuppression treatment (months) and DNA methylation of FoxP3 Treg-specific demethylated region (TSDR). Previous duration of oral immunosuppressive treatment negatively correlated with DNA methylation at FoxP3 TSDR, and this relationship was significant (*P* = 0.012) when modeled with a simple linear regression.

A bivariate analysis of duration of previous oral treatment (months) and CpG methylation at FoxP3 TSDR showed that CpG methylation levels at FoxP3 TSDR negatively correlated with previous duration of oral immunosuppressive treatment (simple linear regression shown in Figure 9). A multivariate linear regression model of methylation of the FoxP3 TSDR, which included the duration of previous oral immunosuppressive treatment (log transformed variable) as a predictor, was significant (*P* = 0.002) and explained around 42% of the variance in FoxP3 TSDR. Additional predictive variables, including TIGIT^+^, FoxP3^+^, IL-10, and TGF-β levels, added stepwise to the multiple regression model were not significant and were, therefore, removed from the final model. Subject age (years) was added to the regression model as a co-variate to adjust for its effect on methylation and the model remained significant (*P* = 0.006), explaining around 41% of the variance in FoxP3 TSDR (Table 4).

**Table 4:** Summary of multiple regression model of methylation of FoxP3 Treg-specific demethylated region.

	Coefficient	*P*-value
Previous duration of oral immunosuppressive treatment (months)	−5.919	0.002**
Age (years)	0.073	0.478

**Significant at the 1% level.

### TIGIT^+^ Tregs Are a Sensitive but Not Specific Biomarker of Clinical Remission in Sight-Threatening Non-Infectious Uveitis

A logistic regression model that included TIGIT^+^ Treg levels and subject age (years) as predictors of clinical remission was significant [χ^2^(2) = 18.402, *P* < 0.001] and a good fit for the data [Hosmer and Lemeshow test; χ^2^(8) = 5.786, *P* = 0.671] (Table 5). It correctly classified 84% subjects and explained around 45% of the variance in clinical remission of sight-threatening non-infectious uveitis. TIGIT was a significant predictor of clinical remission, whereas age was not significant. Peripheral blood TIGIT^+^ Treg levels in the study population of patients with uveitis had a sensitivity of 92%, a specificity of 62%, a false positive rate of 38% and a false negative rate of 8% for detecting clinical remission (Table S4 in Supplementary Material).

**Table 5:** Summary of logistic regression model of clinical remission.

	Odds ratio	95% C.I.	*P*-value
TIGIT^+^ T-regulatory cell	3.1	1.5, 6.5	0.020*
Age (years)	1.0	0.9, 1.1	0.365

*Significant at the 5% level.

## Discussion

In this study, we investigated the hypothesis that phenotypically stable Treg induced by immunosuppressive drugs are associated with sustained clinical remission of non-infectious uveitis. We did this by performing peripheral blood immunophenotyping of large group of patients, who had previously received immunosuppressive drug therapy and were in sustained clinical remission, and comparing these results with those from patients with active uveitis and control subjects. Our data show that the frequency of CD4^+^CD25^+^FoxP3^+^ Treg, TIGIT^+^ Treg, T-bet^+^ Treg and the ratio of CD4^+^CD25^+^FoxP3^+^ Treg to Th1 (T-bet^+^CD4^+^) T-cells are higher in patients in clinical remission compared with patients with active disease. T-bet^+^FoxP3^+^ cell levels showed significant positive correlation with TIGIT^+^FoxP3^+^ cell levels. The levels of CD4^+^FoxP3^+^TIGIT^+^ Treg, CD4^+^CD25^+^FoxP3^+^ Treg and CD4^+^FOXP3^−^TIGIT^+^ T-cells in each subject sample were analyzed as ratios of TIGIT^+^ Treg to TIGIT^+^ T-cells (T-cells which expressed TIGIT but not FoxP3). These ratios were significantly higher in the remission group compared with the active group. This suggested that TIGIT expression proportionally increased in both the CD4^+^FoxP3^+^and the CD4^+^CD25^+^FoxP3^+^ compartment (with the highest levels TIGIT expression in the CD25^+^ subset of FoxP3^+^ cells) of T-cells in the clinical remission group compared with the active group, rather than increasing on all T-cells. This provides some supporting evidence for the utility of TIGIT as a marker of functional Treg, as opposed to being a marker of T-cell activation.

We utilized a novel approach of NGS bisulfate sequencing targeted toward T-cell genes of interest, to identify whether there was an epigenetic immune methylation pattern associated with clinical remission in uveitis. In this study, PBMCs from patients in clinical remission had lower levels of DNA methylation at CpG sites within the FOXP3 TSDR, FOXP3 promoter, and TIGIT loci compared with patients with active disease, supporting the higher levels of FoxP3 and TIGIT expression detected by flow cytometry in these patients. Through *in vitro* functional studies, we demonstrate that Treg from patients in clinical remission, a population in which there were high levels of Treg and TIGIT expression, are effective at suppressing T-cell proliferation. Together, these findings suggest that Treg from patients in clinical remission are polarized toward Type 1 inflammation and have a stable highly suppressive phenotype, characterized by intracellular T-bet and extracellular TIGIT expression, respectively, in comparison with subjects with active disease.

The cytokine milieu in non-infectious uveitis has been previously been investigated by our group and others (42–47). In this study, our focus was on investigating serum levels of specific cytokines associated with the Treg, Th1 and Th17 subsets identified in the flow cytometric analysis. Intracellular cytokine levels of IL-10, IFN-γ, and IL-17A were also evaluated in active disease at 0 and 6 months after starting therapy, at which time, disease had resolved. We found that patients in clinical remission had higher serum levels of IL-10 and TGF-β than patients with active disease. While control subjects also had higher levels of IL-10 than active patients, the higher serum TGF-β differentiated remission patients from controls and were perhaps related to high levels of Treg with suppressive function in this group. Tregs levels positively correlated with serum IL-10 and TGF-β levels, suggesting that Tregs may directly or indirectly influence the systemic cytokine milieu. Furthermore, increased intracellular expression of IL-10 by T-cells from active disease was increased 6 months after starting treatment, which corresponded with clinical resolution of disease.

Subjects’ levels of Th1 and Th17 were evaluated by flow cytometric detection of their respective transcription factors, T-bet and RORγt, along with the corresponding serum cytokine levels of IFN-γ, IL-17, and IL-22 from peripheral blood samples. Upregulated T-bet expression in association with significantly increased IFN-γ levels has been previously demonstrated in patients with active uveitis secondary to Vogt–Koyanagi–Harada (VKH) syndrome, a bilateral chronic granulomatous panuveitis, which tends to be sight-threatening (48). Th17 cells have also been shown to be involved in ocular inflammation (49) and, more recently, B27^+^ anterior uveitis (50). Paradoxical expression of effector CD4 T-cell transcription factors, such as T-bet and RORγt, by Treg has, however, been suggested to enhance Treg suppressive capacity in murine models of inflammation (51–54). These models demonstrate that intestinal RORγt^+^FoxP3^+^ Treg induced *in vivo* by the local microbiota display a stable suppressive phenotype and exist in dynamic balance with pathogenic Th17 (53, 55). The local microenvironment, for example, the cytokine milieu and/or the microbiome, have been shown to regulate the Treg/Th17 balance and influence cell plasticity; disruption of this balance may lead to the development of inflammatory disease (54–57). Furthermore, during Type 1 inflammatory responses, Treg may upregulate T-bet in response to IFN-γ production (51). These T-bet^+^ Treg have been observed to be phenotypically stable and selectively suppressive of Th1 (52). However, it is noted that other studies have demonstrated that T-bet expression is not required for Treg to suppress inflammation, in the context of CNS inflammation and colitis (58).

In this study, we show that in clinical remission and control groups, the levels of T-bet^+^ Treg and ratios of Treg to Th1 are higher and serum levels of IFN-γ levels are lower compared with active uveitis patients. We also found that T-bet^+^ Th1 levels in uveitis patients were overall lower in clinical remission compared with active and control subjects. Disease resolution in active patients over the course of 12 months appeared to be associated with an increase in levels of Treg and a decrease in levels of Th1. Intracellular expression levels of IFN-γ by CD4^+^ T-cells were also decreased at 6 months after starting treatment. However, RORγt^+^ Th17 and RORγt^+^ Treg levels did not significantly differ between the three subject groups. Tregs are thought to regulate the expression of IL-22, a Th17 cytokine which facilitates inflammatory cell infiltration in uveitis (59, 60). Although the ratio of Treg to Th17 was not significantly greater in clinical remission, it was noted that serum IL-17A and IL-22 levels were significantly lower in the patients in clinical remission, compared with active and control subjects. RORγt^+^ Treg levels were increased in all four active patients at 12 months compared with baseline, as the disease clinically resolved. However, unlike Th1 cytokine levels, Th17 cytokine levels did not appear to consistently decrease when disease resolved in active patients. Three out of four active patients had increased serum levels of IL-17A at 12 months after starting treatment, compared with baseline. Intracellular expression levels of IL-17A of by CD4^+^ T-cells in active disease also increased at 6 months after starting treatment. Our results show that Th17 express FoxP3^+^ during disease resolution, in response to immunosuppressive treatment, but suggest that Th17-mediated inflammation is not driving inflammation and that FoxP3^+^RORγt^+^ Tregs are not significantly associated with clinical remission.

It was further investigated whether clinical remission in non-infectious sight-threatening uveitis could be modeled by a linear relationship with predictive immunological variables, after adjusting for co-variates in the model. Multiple regression analysis showed that the duration of previous oral immunosuppressive treatment was the strongest predictor of hypomethylation at the FoxP3 TSDR (with the simple linear regression analysis demonstrating an observable hypomethylation effect of oral immunosuppression at the FoxP3 TSDR over several months of treatment). Hypomethylation at the FoxP3 TSDR has been shown to be important for Treg suppressive function and phenotypic stability and was significantly associated with clinical remission in our analysis. The association between previous duration of oral immunosuppression and methylation at FoxP3 TSDR remained significant after adjusting for subject age. This suggests that the duration of oral immunosuppression is an important factor in determining the phenotypic stability of Treg and supports the role of immunosuppressive drug induced Treg in the maintenance of clinical remission. Logistic regression showed that peripheral blood TIGIT levels, but not subject age, were a significant predictor of clinical remission in sight-threatening non-infectious uveitis. TIGIT^+^ Treg levels had high sensitivity (92%) but low specificity (62%) for clinical remission. This infers a high false positive rate for using TIGIT^+^ Treg as a predictor of clinical remission, that is, a patient could test positive for disease remission when they actually have subclinical active disease. TIGIT^+^ Treg levels detected by flow cytometry explained almost half of the variance observed in this model of clinical remission. However, many other factors not accounted for in this analysis, for example, posttranslational protein modifications and other immune markers such as chemokines, could also influence clinical remission.

Previous studies have compared Treg levels between active uveitis, inactive uveitis and/or healthy control subjects but there has been variation in subject groups and clinical immunophenotyping. A study from Chen et al. included 49 patients with VKH syndrome with uveitis and without uveitis, and showed a decreased percentage of CD4^+^CD25^high^ T-cells, a decreased frequency of Foxp3^+^ expression in CD4^+^CD25^high^ T-cells and reduced functionality of CD4^+^CD25^high^ “Treg” in patients with active uveitis (61). Yeh et al. also demonstrated that patients with active uveitis (*n* = 8) have lower percentages of CD4^+^FoxP3^+^ lymphocytes than patients with inactive disease (*n* = 12) (13). In contrast to this study, several patients in their series demonstrated evidence of systemic autoimmune disease, such as sarcoidosis and multiple sclerosis, which are, of themselves, associated with abnormal Treg populations or deficits in Treg suppressive function. Another study compared Treg levels in active and inactive uveitis patients and found that patients in remission on treatment (*n* = 25) had significantly increased levels of CD4^+^CD25^+^FoxP3^+^ Treg compared with active patients on treatment (*n* = 6) (12). However, over half of the patients (53%) recruited to their study had a diagnosis of anterior uveitis, which is not usually sight-threatening and does not require systemic immunosuppression.

Regarding the comparison of Treg levels in active patients with control subjects, the results from the literature are mixed. Two of aforementioned studies show a higher level of Treg in control subjects compared with uveitis patients (12, 61); however, this could be due to the inclusion of patients with systemic disease in the uveitis groups. By contrast, Yeh et al. comment that they have previously observed that the percentages of CD4^+^FoxP3^+^ lymphocytes do not differ between patients with uveitis and control subjects (unpublished data) (13). Molins et al. compared Treg levels and cytokine production in 21 patients with active non-infectious uveitis with 18 controls (18). They found that PBMCs from uveitis patients produced lower levels of IL-10 than those from controls but no differences were observed in Treg levels. TGF-β levels were not measured and functional assays were not performed in this study. Recently, Zhuang et al. compared 20 patients with active B27^+^ anterior uveitis with healthy controls and observed an increase in CD4^+^IL-17^+^ T cells, a decrease of CD4^+^CD25^+^Foxp3^+^ Treg and a higher ratio of Th17/Treg in peripheral blood of patients compared with controls (50). The authors conclude that the imbalance of Th17 and Treg cells may play a vital role in the pathogenesis of B27^+^ anterior uveitis. Of note, patients with B27^+^ uveitis and anterior uveitis were excluded from this study due to the typically non-sight-threatening anterior anatomic localization of inflammation and systemic disease associations.

There has been interest in glucocorticoid-resistant Th17 cells which are refractory to Treg mediated suppression (62, 63), and these have been previously investigated in sight-threatening uveitis (64). Furthermore, a subset of “non-classic” Th17-derived Th1 have been described, which are demethylated at RORC, and are thought to play a pivotal role in the establishment and persistence of inflammatory disease (65). These non-classic Th1 express CD161, a marker of steroid-resistant human Th17, induced by RORC (66). In the clinical setting, however, most cases of uveitis show a good clinical response to high dose corticosteroids. Second-line immunosuppressive drugs are usually prescribed because the dose of corticosteroids required to suppress disease activity is very high and disease recurs on tapering the dose, rather than because the disease is refractory to corticosteroids. Only one patient in this study received biological therapy (Adalimumab), as the rest were clinically responsive to corticosteroids, with or without addition of conventional steroid-sparing agents. Twenty percent of the clinical remission group received previous therapy with cyclosporine, which has been shown to selectively attenuate steroid-resistant Th17 and to be efficacious in the treatment of steroid-refractory disease (67, 68). It is of note, in this study, that the clinical remission and control groups had significantly higher levels of RORC CpG methylation than the active group. Furthermore, within the active patient cohort, higher RORC% CpG methylation levels were observed at 12 months after starting/changing immunosuppressive treatment, when disease had resolved. RORC CpG hypermethylation could be a biomarker of clinical responsiveness to corticosteroid treatment; however, this requires further investigation.

The findings of this study are consistent with previous studies in uveitis demonstrating that Treg levels are higher and more functional in patients with inactive disease, compared with both patients with active and control subjects. We have previously observed that in ocular Behçet’s disease, peripheral blood Treg levels are increased by 3 months’ treatment with the immunomodulating therapy, pegylated interferon-α (pegIFNα), and that the increased levels of Tregs are still detectable at 12 months, 6 months after cessation of therapy (16). However, the expression of the co-inhibitory molecule TIGIT by Treg from patients in sustained clinical remission of disease, who are no longer on therapy, has not been previously investigated in uveitis. It is known that stable FoxP3 expression is ensured, at least partly, by DNA demethylation at the FOXP3 TSDR and that this infers commitment to the Treg lineage (28–31). In a genome-wide methylation analysis of nTregs from healthy individuals, it was found that hypomethylation at the TIGIT locus was one of the most significantly differentially methylated regions that distinguished naïve T-cells and nTreg; that it was not altered by activation and is required for FoxP3 binding (36). Other studies have demonstrated a key role for TIGIT in Treg-mediated suppression of Th1 and Th17 subsets (35, 37) and that TIGIT signaling in Treg directs their immunoregulatory phenotype in chronic disease settings (69). Our data show that hypomethylation at FOXP3 TSDR, FOXP3 promoter, and TIGIT CpG sites and higher levels of TIGIT^+^ Tregs are associated with clinical remission, and may have value in determining remission, of relapsing and remitting sight-threatening non-infectious uveitis.

Our study addressed a need to define and investigate peripheral biomarkers for sustained clinical and biological disease remission in sight-threatening non-infectious uveitis. The total number of subjects recruited to this study (*n* = 60) represents one of the largest sample sizes of published clinical immunology studies of sight-threatening non-infectious uveitis. To improve the specificity of this study toward detecting peripheral blood biomarkers associated with ocular inflammation, we excluded patients with known systemic diagnoses, for example, sarcoidosis or Behçet’s disease, and any other identifiable systemic inflammation. We were rigorous in our clinical phenotyping of the recruited subjects and undertook a comprehensive immunophenotypic analysis of CD4^+^ T-cells derived from these groups, including functional and epigenetic methylation studies, which has not been previously performed in uveitis patients. Our group has previously analyzed aqueous humor samples from the eyes of patients with uveitis (47). However, the procedure of direct intraocular sampling carries risks of introducing infection, triggering or exacerbating inflammation and causing retinal detachment to the eye being sampled. Even when it is deemed clinically necessary, sequential/repeated intraocular sampling should be avoided. Therefore, T-cell immunophenotyping by flow cytometry was performed on freshly isolated subject PBMCs (within 4 h of venepuncture), but additional intraocular sampling was not undertaken as part of this study. The 37 patients in clinical remission recruited to the study demonstrated a good clinical ocular response to immunosuppressive therapy given systemically, supporting the fact that systemic Treg and their associated microenvironment influence ocular immunity and that peripheral blood biomarkers have utility in monitoring ocular disease. Phenotypic analysis of Treg, Th1, and Th17 subtypes was based on “master transcription factor” expression, with prospective intracellular cytokine analysis only in selected subjects with active disease, followed up over a 12-month period. An in-depth analysis of intracellular cytokine production and methylation at signature cytokine loci could be considered for a future study. The number of Treg functional assays that were performed as part of this study was limited by the remaining PBMC numbers after the immunophenotypic analysis, rendering firm conclusions from these results difficult. The assay findings were in keeping with those from previous studies, which have demonstrated that in the inflammatory setting of autoimmune disease, Treg have reduced suppressive function (11, 70). However, they could be confirmed as part of a further study.

The clinical utility of TIGIT^+^ Treg as a single biomarker for remission is limited by its potentially high false positive rate in this regard. It is possible that the calculated test sensitivity, specificity, false positive and negative rates were skewed by the overweighting of the sample toward remission patients rather than active patients. TIGIT levels explained almost half of the variance observed in the model of clinical remission, but there may be other clinically relevant factors, not accounted for in the model, which were also influencing clinical remission. These would need to be further investigated to develop better models for predicting clinical remission for individual patients, which could augment standard clinical assessment of patients. However, our results suggest that low levels of TIGIT may be a biomarker for increased risk of disease relapse in sight-threatening non-infectious uveitis. This could help to inform clinical management decisions, by indicating whether immunosuppressive drugs have induced functionally suppressive Treg.

In summary, our data show that CD4^+^ T-cells in chronic relapsing, remitting sight-threatening non-infectious uveitis deviate toward the Th1 type and that Treg with upregulated levels of T-bet and TIGIT expression are associated with disease remission. These data provide supporting evidence that Tregs are part of the mechanism of clinical remission in non-infectious uveitis, which has been previously demonstrated in EAU model studies only (7–9). We suggest that the overall lower T-bet expression and IFN-γ levels detected in patients who achieve sustained clinical remission are related to potent suppression of Type 1 inflammation by phenotypically stable Treg induced by systemic immunosuppressive therapy. This is an important finding because a recent study in mice has shown that Treg expressing T-bet have a stable and functional phenotype, which potentiates suppression of Th1 autoimmunity (52), but it has not yet been demonstrated in clinical studies. Our results are also consistent with evidence from EAU that relapsing, remitting uveitic disease is closely associated with a Th1-like phenotype whereas monophasic inflammation is associated with a Th17-like phenotype (71).

Finally, these data are relevant to the recent interest in quantitative identification and isolation of viable, functional Treg for downstream clinical purposes, which include their generation *ex vivo* for immunomodulating therapies (72, 73). The results of this study provide evidence that TIGIT has utility as a marker of functional Treg for these therapies.

## Ethics Statement

This study was carried out in accordance with the recommendations of the UK National Research Ethics Service (NRES)—London Harrow Committee (13/LO/1653; 16039) with written informed consent from all subjects, in accordance with the Declaration of Helsinki. The protocol was approved by the Moorfields Eye Hospital National Health Service (NHS) Foundation Trust Department of Research and Development.

## Author Contributions

RG, SL, VC, RS, XZ, ME, OT-N, DN, GG, CM, and NM contributed to the design of the study and experiments. RG, XZ, RS, MC, and NM performed the experiments, data capture, and analysis. RG, VC, SL, and ME performed the data interpretation. All the authors contributed to the critical revision of the manuscript for intellectual content and final approval of published version and agreed to be accountable for the work.

## Conflict of Interest Statement

This research was conducted in the absence of any commercial or financial relationships that could be construed as a potential conflict of interest.

## Supplementary Material

Figure S1 Bisulfite amplicon epigenetic CpG methylation target sites for FOXP3 TSDR, FOXP3 promoter, TBX21, RORC2, and TIGIT loci.

Table S1 Kruskal–Wallis *post-hoc* test pairwise comparisons of immunological markers across the three subject groups.

Table S2 Primer sequences used in the Fluidigm assay for DNA methylation analysis (the Fluidigm CS1 and CS2 tags are highlighted in bold).

Table S3 Unique Fluidigm barcodes added to the PCR products in the Fluidigm assay for DNA methylation analysis.

Table S4 TIGIT^+^ Treg as a biomarker of clinical remission.
