# Supplementary figures and images for: Clinical Remission of Sight-Threatening Non-Infectious Uveitis Is Characterized by an Upregulation of Peripheral T-Regulatory Cell Polarized Towards T-bet and TIGIT
Source: Front Immunol. 2018 May 3;9:907. doi: 10.3389/fimmu.2018.00907 (PMC5943505; doi:10.3389/fimmu.2018.00907)

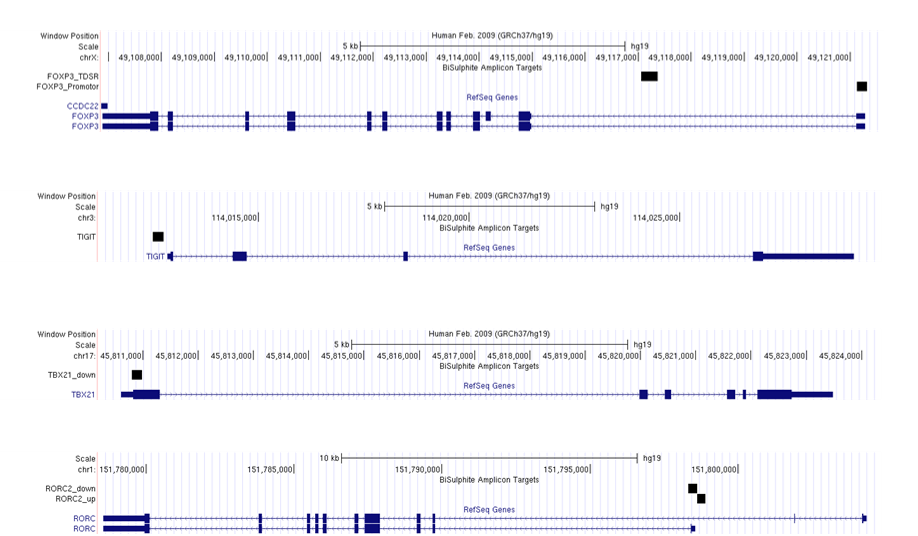

Supplement: Figure S1 — Bisulfite amplicon epigenetic CpG methylation target sites for FOXP3 TSDR, FOXP3 promoter, TBX21, RORC2, and TIGIT loci. [file Image_1.tif]
