# Supplementary material for: Clinical Remission of Sight-Threatening Non-Infectious Uveitis Is Characterized by an Upregulation of Peripheral T-Regulatory Cell Polarized Towards T-bet and TIGIT
Source: Front Immunol. 2018 May 3;9:907. doi: 10.3389/fimmu.2018.00907 (PMC5943505; doi:10.3389/fimmu.2018.00907)
Supplement: Table S1 — Kruskal–Wallis post-hoc test pairwise comparisons of immunological markers across the three subject groups. [file Table_1.docx]

| Immunological Markers | Summary P-value  (Kruskall-Wallis) | | | Adjusted P-values  (Post-hoc Pairwise Comparison Between  Remission, Active and Control Subjects) | | |  |
| --- | --- | --- | --- | --- | --- | --- | --- |
|  | | |  | **Remission**  **v.**  **Active** | **Remission**  **v.**  **Control** | **Active**  **v.**  **Control** |  |
| **T-cell subsets (%) and ratios in the**  **CD3^+^CD4^+^ compartment** | |  | |  |  |  |  |
| **Treg (CD25^+^FoxP3^+^)** | | 0.000*** | | ↑ 0.000*** | 0.118 | 0.267 | |
| **TIGIT^+^Treg** | | 0.000*** | | ↑ 0.000*** | 0.661 | 0.126 | |
| **TIGIT^+^Treg: TIGIT^+^FoxP3^-^** | | 0.049* | | ↑ 0.049* | 1.000 | 0.219 | |
| **TIGIT^+^FoxP3^+^CD25^+^: TIGIT^+^FoxP3^-^** | | 0.003** | | ↑0.002** | 0.111 | 0.091 | |
| **Th1 (Tbet^+^)** | | 0.025* | | ↓ 0.024* | 0.755 | 0.858 | |
| **Th17 (RORγt^+^)** | | 0.564 | | - | - | - | |
| **Treg: Th1** | | 0.001** | | ↑ 0.001** | 1.000 | ↓ 0.013* | |
| **Treg: Th17** | | 0.000*** | | 0.828 | ↑ 0.000*** | ↑ 0.014* | |
| **Tbet^+^Treg** | | 0.000*** | | ↑ 0.005** | ↑ 0.000*** | 0.859 | |
| **RORγt^+^Treg** | | 0.363 | | - | - | - | |
| **Th1/17 (RORγt^+^ Tbet^+^ )** | | 0.183 | | - | - | - | |
| **Serum cytokine levels (pg/mL)** | |  | |  |  |  | |
| **IL-10** | | 0.000*** | | ↑ 0.011* | 0.082 | ↓ 0.000*** | |
| **TGF-β** | | 0.001** | | ↑ 0.007** | ↑ 0.004** | 1.000 | |
| **IFN-γ** | | 0.000*** | | ↓ 0.015* | 0.130 | ↑ 0.000*** | |
| **IL-17** | | 0.003** | | ↓ 0.002** | 0.460 | 0.408 | |
| **IL-22** | | 0.002** | | ↓ 0.001** | 0.693 | 0.210 | |
| **CpG site methylation levels (%)** | |  | |  |  |  | |
| **FOXP3 Promoter** | | 0.037* | | ↓ 0.036* | 0.405 | 1.000 | |
| **FOXP3 TSDR** | | 0.005** | | ↓ 0.003** | 0.709 | 0.177 | |
| **TIGIT** | | 0.004** | | ↓ 0.003** | 0.850 | 0.120 | |
| **TBX21/ TBET** | | 0.122 | | - | - | - | |
| RORC2/ RORγT | | 0.006* | | ↑ 0.016* | 1.000 | ↓ 0.014* | |
