# Supplementary material for: Clinical Remission of Sight-Threatening Non-Infectious Uveitis Is Characterized by an Upregulation of Peripheral T-Regulatory Cell Polarized Towards T-bet and TIGIT
Source: Front Immunol. 2018 May 3;9:907. doi: 10.3389/fimmu.2018.00907 (PMC5943505; doi:10.3389/fimmu.2018.00907)
Supplement: Table S2 — Primer sequences used in the Fluidigm assay for DNA methylation analysis (the Fluidigm CS1 and CS2 tags are highlighted in bold). [file Table_2.docx]

**PCR cycling conditions: MJ Research PTC-225 Peltier Thermal Cycler was used for all PCR cycling conditions.*

*PCR Conditions: 95°C for 1min;[ 95°C for 15seconds, ‘Annealing temp’ see table for 15 seconds, 72° C for 10 seconds] cycle 35 times; 4°C hold*
