# Supplementary material for: Clinical Remission of Sight-Threatening Non-Infectious Uveitis Is Characterized by an Upregulation of Peripheral T-Regulatory Cell Polarized Towards T-bet and TIGIT
Source: Front Immunol. 2018 May 3;9:907. doi: 10.3389/fimmu.2018.00907 (PMC5943505; doi:10.3389/fimmu.2018.00907)
Supplement: Table S3 — Unique Fluidigm barcodes added to the PCR products in the Fluidigm assay for DNA methylation analysis. [file Table_3.docx]

| **Fluidigm Barcode sequences (5’-3’)** |
| --- |
| TGGTACTCGC |
| GAAGTCGCTG |
| GCGTTGTGGT |
| CGTCGTAGCT |
| TGTGATCTGG |
| CGGTGGTGAT |
| CGCTGGTACA |
| GAGGTCTAGT |
| GAACGTGTCG |
| CTGGAGCGAT |
| TTCGTCTCAG |
| GTTAGCTTCG |
| TTGGCGTGTG |
| TGATCGCCGT |
| GATGGCTAGA |
| TGCCGCTGTT |
| TTGTTGCGCC |
| GGAGCGATCA |
| GTCGAGGATC |
| TGTCTACTGG |
| GTTGTACTGC |
| GGCTCTGGTA |
| TGCAGGCTGT |
| GTCACGTGGT |
| GTAGGATCGG |
| TCGCTGATGG |
| GCTTAGTCAC |
| CTGGACATCT |
| TACGGAGGTA |
| CGTAGAGAGG |
| GCTCGATCTT |
| GTGCCAGAGT |
| CTTGGTGCTG |
