# Supplementary material for: Clinical Remission of Sight-Threatening Non-Infectious Uveitis Is Characterized by an Upregulation of Peripheral T-Regulatory Cell Polarized Towards T-bet and TIGIT
Source: Front Immunol. 2018 May 3;9:907. doi: 10.3389/fimmu.2018.00907 (PMC5943505; doi:10.3389/fimmu.2018.00907)
Supplement: Table S4 — TIGIT+ Treg as a biomarker of clinical remission. [file Table_4.docx]

| **Supplementary Table 4 TIGIT^+^ Treg as a biomarker of clinical remission**   \| **TIGIT** \| **Observed - Clinical Remission** \| **Observed - Active Disease** \| \| --- \| --- \| --- \| \| **Predicted - Clinical Remission** \| 34 (TP) \| 5 (FP) \| \| **Predicted –**  **Active Disease** \| 3 (FN) \| 8 (TN) \|   where TP=true positive, FP= false positive, FN= false negative, TN=true negative |
| --- | --- | --- | --- | --- | --- | --- | --- | --- | --- |
